# Clonal inactivation of TERT impairs stem cell competition

**DOI:** 10.1038/s41586-024-07700-w

**Published:** 2024-07-17

**Authors:** Kazuteru Hasegawa, Yang Zhao, Alina Garbuzov, M. Ryan Corces, Patrick Neuhöfer, Victoria M. Gillespie, Peggie Cheung, Julia A. Belk, Yung-Hsin Huang, Yuning Wei, Lu Chen, Howard Y. Chang, Steven E. Artandi

**Affiliations:** 1 Stanford Cancer Institute, Stanford University School of Medicine, Stanford, CA USA; 2 Department of Medicine, Stanford University School of Medicine, Stanford, CA USA; 3 Department of Biochemistry, Stanford University School of Medicine, Stanford, CA USA; 4 Center for Personal Dynamic Regulomes, Stanford, CA USA; 5 Nuclear Dynamics and Cancer Program, Cancer Epigenetics Institute, Fox Chase Cancer Center, Philadelphia, PA USA; 6 Department of Computer Science, Stanford University, Stanford, CA USA; 7 Howard Hughes Medical Institute, Stanford University, Stanford, CA USA

**Keywords:** Adult stem cells, Ageing, Tumour heterogeneity

## Abstract

Telomerase is intimately associated with stem cells and cancer, because it catalytically elongates telomeres—nucleoprotein caps that protect chromosome ends^1^. Overexpression of telomerase reverse transcriptase (TERT) enhances the proliferation of cells in a telomere-independent manner^2–8^, but so far, loss-of-function studies have provided no evidence that TERT has a direct role in stem cell function. In many tissues, homeostasis is shaped by stem cell competition, a process in which stem cells compete on the basis of inherent fitness. Here we show that conditional deletion of *Tert* in the spermatogonial stem cell (SSC)-containing population in mice markedly impairs competitive clone formation. Using lineage tracing from the *Tert* locus, we find that TERT-expressing SSCs yield long-lived clones, but that clonal inactivation of TERT promotes stem cell differentiation and a genome-wide reduction in open chromatin. This role for TERT in competitive clone formation occurs independently of both its reverse transcriptase activity and the canonical telomerase complex. Inactivation of TERT causes reduced activity of the MYC oncogene, and transgenic expression of MYC in the TERT-deleted pool of SSCs efficiently rescues clone formation. Together, these data reveal a catalytic-activity-independent requirement for TERT in enhancing stem cell competition, uncover a genetic connection between TERT and MYC and suggest that a selective advantage for stem cells with high levels of TERT contributes to telomere elongation in the male germline during homeostasis and ageing.

## Abstract (web summary)

Studies in mice show that telomerase reverse transcriptase (TERT) has a role in enhancing stem cell competition that is independent of its reverse transcriptase activity, and promotes chromatin accessibility and activity of the MYC oncogene.

## Main

Telomerase is enriched in tissue stem cells and activated in many cancers by somatic TERT promoter mutations^9–11^. The core of the telomerase enzyme comprises the catalytic subunit TERT and the telomerase RNA component *Terc*—a small non-coding RNA scaffold that encodes the template for telomere addition^1^. The crucial requirement for telomerase in long-term cell viability is conserved from single-cell eukaryotes to humans^12,13^. Cell proliferation in the absence of telomerase results in a lag phase that is at first well tolerated, while telomere reserves are ample, but which culminates in senescence or cell death as telomeres become short and dysfunctional. In laboratory mice with very long telomeres (40–80 kb, as compared with 5–15 kb in humans), germline inactivation of *Tert* or *Terc* results in viable mice, but subsequent intergenerational breeding is accompanied by progressive telomere shortening, which results in severe tissue defects owing to critically short telomeres after six generations^14–16^. In contrast with these loss-of-function studies, overexpression studies have revealed non-canonical functions of TERT, which were separable from telomere synthesis because they also occurred either with a catalytically inactive *Tert* allele or in mice lacking *Terc* (refs. ^3,4,8^). In this context, TERT promoted proliferation or impaired apoptosis, and was shown to activate the MYC, WNT and NF-κB pathways^2–8^. Many renewing tissues are characterized by stem cell competition, a mechanism that optimizes tissue function by promoting the replacement of less-fit stem cells by more-robust neighbouring stem cells^17–21^. This competitive behaviour is also characteristic of carcinogenesis, during which oncogenic mutations drive cells to clonally expand their territory through a process of super-competition^22^. Among tissues in which competitive repopulation has been observed, the testis shows high telomerase activity and exhibits unusual telomere dynamics in that telomere lengths are preserved with ageing, in contrast with the progressive shortening that is seen in other human tissues^23,24^. We previously found that SSCs express high levels of TERT and that TERT is downregulated with lineage commitment^16^. Here, to investigate the telomere-independent role of TERT in stem cells, we developed a system to mark single SSCs expressing TERT, coupled with the ability to conditionally inactivate TERT using a lineage-tracing approach, and examined how clonal TERT deletion affects stem cell competition.

## TERT expression in spermatogenesis

**Extended Data Fig. 1:**
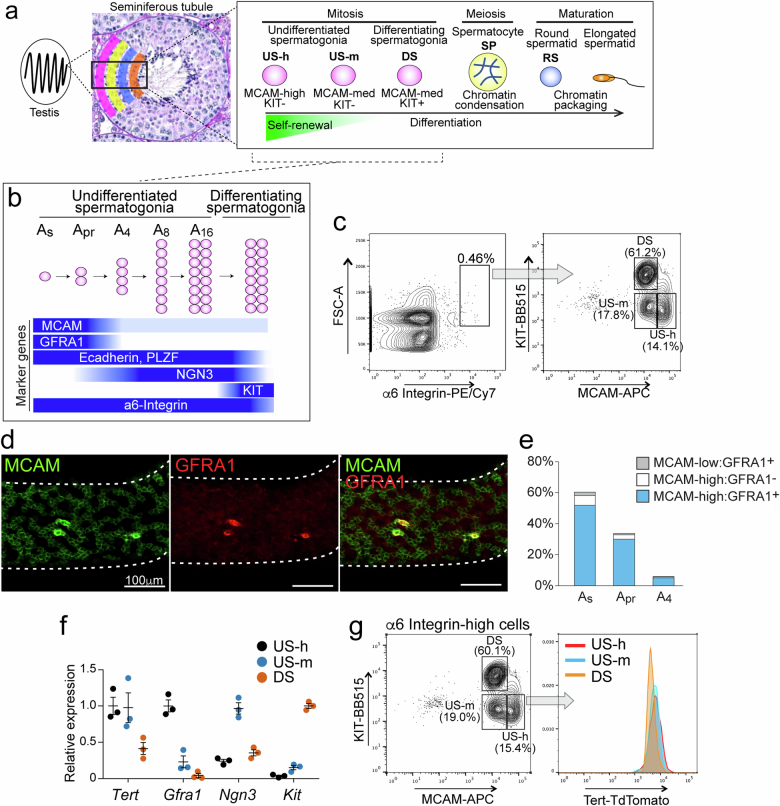
TERT expression in spermatogonia subpopulations. **a**, Spermatogenesis takes place within seminiferous tubules, which are composed of four layers of male germ cells and supporting somatic cells. SSC activity resides in undifferentiated spermatogonia, an immature cell population that undergo self-renewal/mitosis to produce differentiating daughter cells. Undifferentiated spermatogonia can be divided into US-h and US-m based on MCAM expression. US-h are mainly composed of GFRA1^+^ A_s_ and A_pr_ cells that show higher stem cell potential than longer chained US. US-m are comprised of A_al_ cells that are more likely to differentiate into DS than shorter chained cells. DS are KIT+ differentiation-commited progenitor cells that further proliferate and differentiate into meiotic spermatocytes and then to spermatids that undergo nuclear elongation. **b**, A morphological and gene expression heterogeneity in spermatogonia subpopulations. MCAM and GFRA1 are strongly expressed in A_s_ and A_pr_ cells while NGN3 is mainly expressed in A_al_ cells. E-Cadherin and PLZF are expressed in the entire populations of undifferentiated spermatogonia and an early stage of DS. KIT expression is activated when they differentiate into DS. A6-Integrin is expressed from A_s_ to DS. **c**, Flow cytometry analysis of wild-type testicular cells stained with α6-Integrin, MCAM, and KIT. α6-Integrin-high cells were further separated into US-h, US-m, and DS based on KIT and MCAM expression. **d**, Whole-mount immunofluorescence of GFRA1 and MCAM in seminiferous tubules. Scale bars, 100 μm. **e**, Quantification of GFRA1^+^ and MCAM-high cells in seminiferous tubules (n = 262, 146, and 26 chains of interconnected US from 4 mice from left to right). **f**, qRT–PCR of *Tert* and spermatogonia markers in FACS-sorted US-h, US-m, and DS. Expression was normalized with *Actb* (n = 3 mice per group). Data are represented as mean ± s.e.m. **g**, Flow cytometry analysis of testicular cells from *Tert*^*Tdtomato/+*^ mouse. α6-Integrin-high cells were gated and further separated according to KIT and MCAM expression. *Tert-Tdtomato* expression was compared in US-h, US-m and DS.

Spermatogenesis is a dynamic process through which SSCs give rise to sperm through hierarchical mitotic divisions, meiosis and post-meiotic maturation (Extended Data Fig. 1a,b). In the testis, SSCs reside in a functionally and morphologically heterogeneous population termed undifferentiated spermatogonia (US). Singly isolated A_single_ (A_s_) US undergo incomplete cytokinesis, subsequently producing progressively elongating chains of A_paired_ (A_pr_: 2 connected US) and A_aligned_ (A_al_: 4 (A_4_), 8 (A_8_) or 16 (A_16_) connected US)^25^ (Extended Data Fig. 1b). Maturation of US yields differentiating spermatogonia (DS)—committed progenitors that lack stem cell potential. To measure *Tert* expression in distinct subpopulations of spermatogonia, we purified MCAM^high^KIT^−^ US (US-h), MCAM^med^KIT^−^ US (US-m) and MCAM^med^KIT^+^ DS (Extended Data Fig. 1a–c), as we reported previously^26^. Strong expression of MCAM closely overlapped with the expression of GFRA1, a marker enriched in A_s_ and A_pr_ cells (Extended Data Fig. 1d,e). Among these subpopulations, *Tert* mRNA expression was high in both US-h and US-m cells, and decreased in DS cells (Extended Data Fig. 1f). Similarly, in *Tert-Tdtomato* knock-in reporter mice, both US-h and US-m showed high tdTomato expression (Extended Data Fig. 1g). These data indicate that TERT expression is enriched in the entire population of US cells.

## Clone formation from TERT^+^ SSCs

**Fig. 1:**
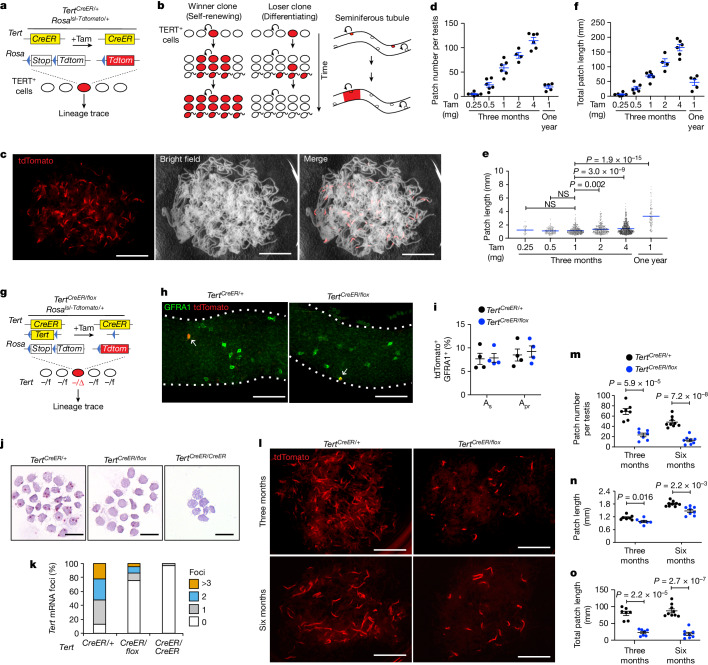
Deletion of TERT impairs SSC-mediated clone formation. **a**, *Tert*^*CreER/*+^*Rosa*^*lsl-Tdtomato/*+^ mice. Tam, tamoxifen. **b**, Lineage tracing using *Tert-CreER*. **c**, Epifluorescence of tdTomato and bright-field image of untangled seminiferous tubules in a single whole testis at three months after the injection of 1 mg tamoxifen. Scale bars, 5 mm. **d**–**f**, Mean patch number (**d**) (*n* = 6, 6, 6, 4, 6, 5 mice from left to right), mean patch length (**e**) (*n* = 6, 6, 6, 4, 6, 5 mice from left to right) and total patch length (**f**) (*n* = 30, 140, 351, 337, 687, 98 patches from left to right) after pulse labelling with the indicated dose of tamoxifen for the indicated time. *P* values determined by one-way ANOVA with Tukey’s test. NS, not significant. **g**, *Tert*^*CreER/flox*^*:Rosa*^*lsl-Tdtomato/+*^ mice. **h**, Immunofluorescence of GFRA1 and tdTomato (day 2) using whole-mount seminiferous tubules. Arrows indicate tdTomato^+^GFRA1^+^ cells. Scale bars, 100 μm. **i**, Quantification of tdTomato^+^ cells among GFRA1^+^ A_s_ and A_pr_ cells (*n* = 4 mice per group). **j**, In situ hybridization against *Tert* mRNA in purified tdTomato^+^ US at five days after tamoxifen treatment. US from *Tert*^*CreER/CreER*^ mice were used as a negative control. Scale bars, 40 μm. **k**, Quantification of foci of *Tert* mRNA (*n* = 232, 242, 341 cells, 3 mice per group, from left to right). **l**, Epifluorescence of tdTomato in untangled seminiferous tubules at three months and six months after tamoxifen injection. Scale bars, 5 mm. **m**–**o**, Quantification of mean patch number (**m**), mean patch length (**n**) and total patch length (**o**) (*n* = 7, 7, 9, 8 mice from left to right). *P* values determined by two-sided unpaired *t*-test. Data are mean ± s.e.m.

To functionally study TERT-expressing spermatogonia, we developed a lineage-tracing assay using *Tert*^*CreER/+*^;*Rosa26*^*lsl-Tdtomato/+*^ (*Tert*^*CreER/+*^) mice, in which TERT-expressing cells are permanently labelled after tamoxifen-dependent activation of CreER to express tdTomato^27,28^ (Fig. 1a,b). Marking SSCs results in long-lived tdTomato^+^ clones, also known as patches, which are composed of many daughter cells produced by the labelled SSC^29^. Conversely, if committed progenitor cells are labelled, only a small transient clone is generated and these cells are lost through differentiation (Fig. 1b). Sparse labelling allows rare SSCs to be marked, and in this context each patch derives from a single SSC. Thus, measuring the number of tdTomato^+^ patches allows a quantitative assessment of stem cell self-renewal activity.

**Extended Data Fig. 2:**
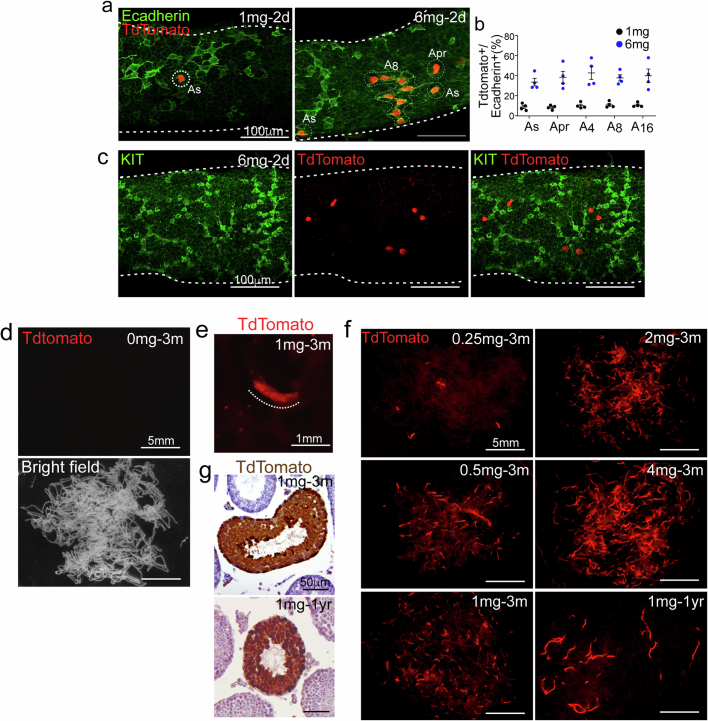
Marking and lineage tracing of TERT^+^ cells. **a**, Whole-mount immunofluorescence of tdTomato and E-cadherin, a membrane marker of US, in whole-mount seminiferous tubules at day two. Scale bars, 100 μm. **b**, Quantification of tdTomato^+^ cells in E-cadherin^+^ US (n = 4 mice per group). Data are represented as mean ± s.e.m. **c**, Whole-mount immunofluorescence of KIT and tdTomato in a *Tert*^*CreER/+*^*:Rosa*^*lsl-Tdtomato/+*^ seminiferous tubule at two days after high dose tamoxifen injection. Scale bars, 100 μm. **d**, Epifluorescence of tdTomato and bright-field image of untangled seminiferous tubules in a single whole testis at 3-month after oil injection. Scale bars, 5 mm. **e**, Singly isolated tdTomato^+^ patch. Scale bar, 1 mm. **f**, Epifluorescence for tdTomato in untangled seminiferous tubules at 3-month or 1-year after pulse labelling; injected tamoxifen dose are shown (0.25–4 mg). Scale bars, 5 mm. **g**, Cross-sections of testes immunostained with antibodies to tdTomato at 3-month or 1-year after labelling. Scale bars, 50 μm.

At two days after tamoxifen injection, tdTomato was detected similarly throughout the population of US cells, but not in KIT^+^ cells (Extended Data Fig. 2a–c). At three months, marking TERT^+^ cells yielded labelled patches, whereas no-tamoxifen controls did not produce patches (Fig. 1c and Extended Data Fig. 2d,e). As we varied the dose of administered tamoxifen from 0.25 mg to 4 mg, patch number increased in a dose-dependent manner (Fig. 1d and Extended Data Fig. 2f). Patch length remained constant at doses lower than 1 mg but increased for those higher than 2 mg, reflecting the fusion of two independently labelled clones with high dose (Fig. 1e and Extended Data Fig. 2f). In mice that were treated with 1 mg tamoxifen and traced for one year, patch length increased, clone number decreased and the total aggregated tdTomato^+^ patch length (mean patch number times mean patch length) remained constant, compared with those traced for three months, consistent with previously described stochastic competition with unlabelled clones^29^ (Fig. 1d–f and Extended Data Fig. 2f). At both three months and one year, patches were composed of cells throughout the spermatogenic lineage (Extended Data Fig. 2g). Together, these data show that TERT-expressing SSCs generate long-lived clones and exhibit competition within the stem cell pool.

## Impaired clone formation with TERT loss

**Extended Data Fig. 3:**
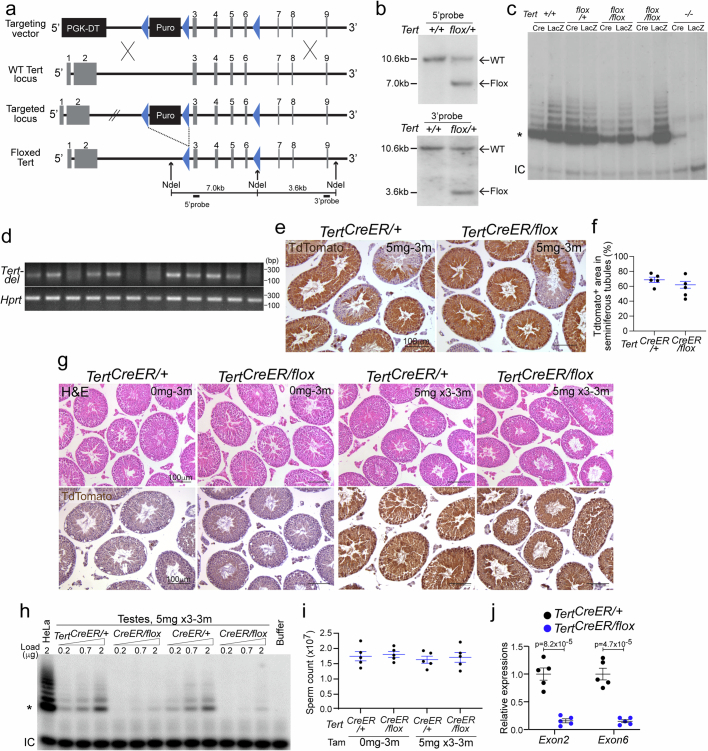
Conditional deletion of TERT in SSCs using *Tert-flox* mice. **a**, *Tert-flox* targeting strategy and Southern blot strategy. **b**, Southern blot analysis of genomic DNA from mouse tails. Locations of 5’ and 3’ probes are shown in **a**. **c**, TRAP assay using mouse embryonic fibroblasts from *Tert-flox* mice. Cells were transfected with adenovirus to express LacZ or Cre. *, first telomerase addition product; IC, internal PCR control. **d**, Genotyping of tdTomato^+^ patches at 3-month after labelling. Primers to detect recombined *Tert* allele that lacks exon 3-6 were used. *Hprt* was used as a positive control. **e**, Immunostaining against tdTomato using testes at 3-month after high dose tamoxifen administration. Scale bars, 100 μm. **f**, Quantification of tdTomato^+^ area in seminiferous tubules at 3-month after high dose tamoxifen administration (n = 5 mice per group). Scale bars, 100 μm. **g**, Histology and immunostaining against tdTomato in *Tert*^*CreER/flox*^ mice at 3-month after oil or tamoxifen administration. **h**, TRAP assay using whole testes at 3-month after 5 mg x 3 times tamoxifen administration. *, first telomerase addition product; IC, internal PCR control. **i**, Sperm count in epididymis at 3-month after labelling (n = 5 mice per group). **j**, qRT–PCR analysis on exon 2 and 6 of *Tert* mRNA in whole testes at 3-month after 5 mg × 3 times tamoxifen administration. Expression was normalized with *Actb* (n = 5 mice per group). *P* values determined by two-sided unpaired *t*-test. Data are represented as mean ± s.e.m.

To investigate a direct role for TERT in SSCs, we developed a competitive clone formation assay using *Tert*^*CreER/flox*^*:Rosa*^*lsl-Tdtomato/+*^ (*Tert*^*CreER/flox*^) mice (Fig. 1g and Extended Data Fig. 3a–c). In this strain, activation of CreER induces tdTomato labelling and concomitantly inactivates *Tert* in the same TERT^+^ cell. Sparse labelling using this strain enables one to trace the fate of cell clones deriving from SSCs in which *Tert* has been somatically deleted in an environment in which most neighbouring cells retain TERT expression. At two days after treatment with 1 mg tamoxifen, GFRA1^+^ A_s_ and A_pr_ clones were marked indistinguishably in both *Tert*^*CreER/+*^ and *Tert*^*CreER/flox*^ mice (Fig. 1h,i). Pulse labelling efficiently eliminated *Tert* mRNA in tdTomato^+^ US (Fig. 1j,k). At three months and six months, deletion of TERT caused a marked reduction in the number of tdTomato^+^ clones and diminished the mean patch length (Fig. 1l–n). Correspondingly, the total aggregated tdTomato^+^ patch length was sharply reduced (Fig. 1o). Genotyping of tdTomato^+^ patches at three months revealed that 36.2% (34/94) of clones did not fully recombine the *Tert* allele (Extended Data Fig. 3d), indicating an enrichment of patches that retain TERT. At three months after injection with higher doses of tamoxifen (5 mg), tdTomato^+^ areas in seminiferous tubules remained unchanged, suggesting that the elimination of TERT-deleted SSCs is alleviated in a less competitive environment (Extended Data Fig. 3e,f). To further differentiate between a role for TERT in stem cell competition versus a requirement for TERT in SSC function, mice were treated with repeated high doses of tamoxifen (5 mg for three consecutive days) and analysed after a three-month trace. tdTomato expression was detected in all seminiferous tubules, and telomerase activity was sharply reduced, whereas spermatogenesis proceeded normally (Extended Data Fig. 3g–i). This indicates that in this context of quantitative deletion throughout all US cells, TERT is not essential for SSC maintenance, which is consistent with results from first-generation germline *Tert*-knockout mice^16^. *Tert* mRNA analysed by quantitative reverse transcription PCR (qRT–PCR) at both exon 2 and exon 6 was markedly reduced, which indicates that transcripts from the *Tert-flox* allele are lost by nonsense-mediated decay after Cre-dependent recombination, precluding notable expression of a truncated protein from the recombined allele (Extended Data Fig. 3j). Together, these findings indicate that focal deletion of TERT in a subset of SSCs compromises clone formation through a process of competition.

## A role independent of catalytic activity

**Fig. 2:**
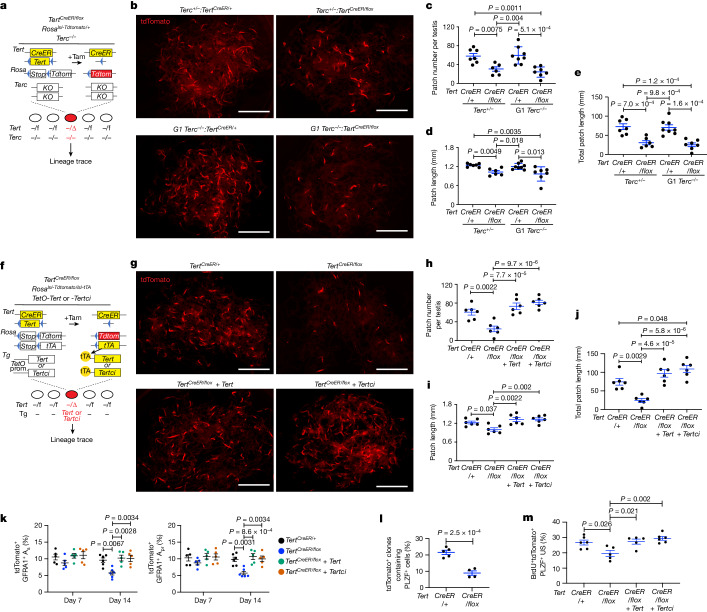
TERT promotes SSC competition independent of the telomerase complex and TERT catalytic activity. **a**, *Tert*^*CreER/flox*^*:Rosa*^*lsl-Tdtomato/+*^*:Terc*^−/−^ mice. **b**, Epifluorescence for tdTomato in untangled seminiferous tubules at three months after injection. Scale bars, 5 mm. **c**–**e**, Quantification of mean patch number (**c**), mean patch length (**d**) and total patch length (**e**) (*n* = 7, 6, 8, 7 mice from left to right). *P* values determined by one-way ANOVA with Tukey’s test. **f**, *Tert*^*CreER/flox*^*:Rosalsl-Tdtomato/lsl-tTA:TetO-Tert or -Tertci* mice. **g**, Epifluorescence for tdTomato in untangled seminiferous tubules at three months after tamoxifen injection. Scale bars, 5 mm. **h**–**j**, Quantification of mean patch number (**h**), mean patch length (**i**) and total patch length (**j**) (*n* = 6 mice per group). *P* values determined by one-way ANOVA with Tukey’s test. **k**, Quantification of tdTomato^+^GFRA1^+^ A_s_ and A_pr_ cells at 7 or 14 days after labelling (*n* = 5, 5, 5, 5, 6, 6, 5, 5 mice from left to right). *P* values determined by one-way ANOVA with Tukey’s test. **l**, Quantification of the percentage of tdTomato^+^ clones containing PLZF^+^ cells at six weeks after tamoxifen injection (*n* = 5, 4 mice from left to right). *P* values determined by two-sided unpaired *t*-test. **m**, Quantification of BrdU^+^ cells in tdTomato^+^PLZF^+^ US at seven days after tamoxifen injection (*n* = 6, 5, 5, 6 mice from left to right). *P* values determined by one-way ANOVA with Tukey’s test. Data are mean ± s.e.m.

**Extended Data Fig. 4:**
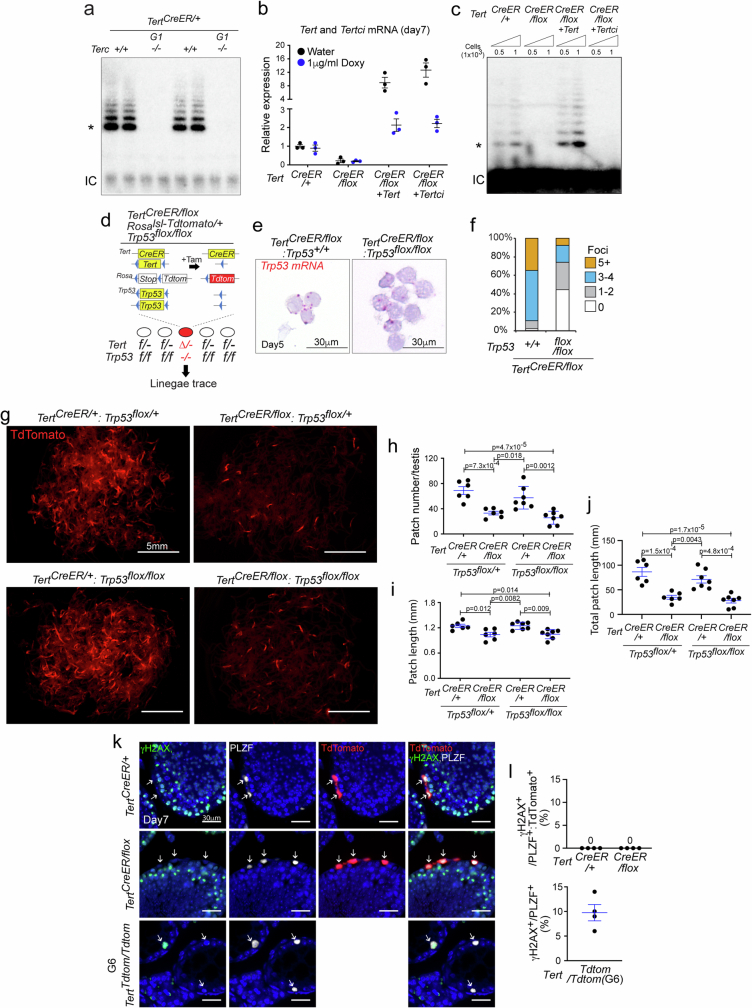
TERT promotes competitive clone formation of SSCs independent of its enzyme activity and the canonical telomerase complex. **a**, TRAP assay using whole testes from *Tert*^*CreER/+*^*:Terc*^*+/+*^ and G1 *Tert*^*CreER/+*^*:Terc*^*−/−*^ mice. *, first telomerase addition product; IC, internal PCR control. **b**, qRT–PCR analysis of *Tert* and *Tertci* in purified tdTomato^+^ US at day 7. Doxycycline was added to drinking water to reduce transgenic *Tert* and *Tertci* expression. Expression was normalized with *Actb* (n = 3 mice per group). **c**, TRAP assay using purified tdTomato^+^ US from indicated genotypes. *, first telomerase addition product; IC, internal PCR control. **d**, *Tert*^*CreER/flox*^*:Rosa*^*lsl-Ttomato/+*^*:Trp53*^*flox/flox*^ mice. **e**, In situ hybridization against *Trp53* mRNA. tdTomato^+^ US were sorted out at 5 days post labelling and stained. **f**, Quantification of foci number of *Trp53* mRNA (n = 174 and 135 cells from 4 mice per group from left to right). **g**, Epifluorescence for tdTomato in untangled seminiferous tubules at 3-month after labelling. Scale bars, 5 mm. **h**–**j**, Quantitative data of patch number (**h**), average patch length (**i**) and total patch length (**j**) (*n* = 6, 6, 7, 7 mice from left to right). *P* values determined by one-way ANOVA with Tukey’s test. **k**, Immunofluorescence of γH2AX, PLZF, and tdTomato in testicular cross-sections. G6 *Tert*^*Tdtomato/Tdtomato*^ mice were used as positive control for γH2AX staining. Normal spermatocytes are positive for γH2AX. Scale bars, 30 μm. **l**, Quantification of γH2AX^+^ PLZF^+^ US (n = 4 mice per group). Data are represented as mean ± s.e.m.

To determine whether the effect of TERT in enhancing SSC competition depends on its catalytic role in elongating telomeres, we performed several studies. *Terc* encodes the RNA template for telomere addition and serves as the central scaffold for assembly of the telomerase complex. Therefore, in the absence of *Terc*, TERT and the other components of telomerase do not associate^1,30^. To determine whether formation of the telomerase complex is required for TERT-dependent SSC clone formation, we produced first-generation *Terc*^−/^^−^:*Tert*^*CreER/flox*^*:Rosa*^*lsl-Tdtomato/+*^ (G1 *Terc*^−^^/^^−^:*Tert*^*CreER/flox*^) mice (Fig. 2a). In this strain, telomerase was inactive in the testes, consistent with disruption of the complex through *Terc* deletion (Extended Data Fig. 4a). Despite the absence of telomerase activity in these mice, conditional deletion of *Tert* in *Terc*-deficient mice impaired patch number, patch length and total patch length when analysed three months after tamoxifen treatment (Fig. 2b–e). These findings indicate that SSCs depend on TERT for effective stem cell competition even in mice lacking telomerease, thereby uncoupling the requirement for TERT in stem cell competition from engagement in the classical telomerase complex.

To understand whether the reverse transcriptase activity of TERT is required for stem cell compeitition, we developed a system to rescue the defect in TERT-deleted cells using tetracycline-regulated TERT transgenes: either wild-type (TetO-TERT) or catalytically inactive (TetO-TERTci) owing to a single amino acid substitution in the catalytic site^8^. We produced compound mouse strains in which activation of CreER simultaneously deletes the floxed *Tert* gene while inducing the expression of transgenic *Tert* by triggering the expression of the tetracycline transactivator (tTA) that binds to and activates the *TetO* promoters (Fig. 2f). We examined how TERT loss affected clone formation in *Tert*^*CreER/+*^*:Rosa*^*lsl-Tdtomato/lsl-tTA*^ (*Tert*^*CreER/+*^) mice versus *Tert*^*CreER/flox*^*:Rosa*^*lsl-Tdtomato/lsl-tTA*^ (*Tert*^*CreER/flox*^) controls, and we compared these results with the restoration of TERT expression in *Tert*^*CreER/flox*^*:Rosa*^*lsl-Tdtomato/lsl-tTA*^:*TetO-Tert* (*Tert*^*CreER/flox*^ *+* *Tert*) mice or *Tert*^*CreER/flox*^*:Rosa*^*lsl-Tdtomato/lsl-tTA*^:*TetO-Tertci* (*Tert*^*CreER/flox*^ *+* *Tertci*) mice (Fig. 2f). Transgenic *Tert* and *Tertci* expression were induced at a physiological range by treating mice with a low dose of doxycycline, which suppresses the binding of tTA to the TetO promoter (Extended Data Fig. 4b). Transgenic expression of wild-type *Tert*, but not *Tertci*, restored telomerase activity (Extended Data Fig. 4c). Notably, expression of either *Tert* or *Tertci* transgenes fully rescued patch number, average patch length and total patch length (Fig. 2g–j). Furthermore, expression of TERTci significantly increased total patch length, compared with control *Tert*^*CreER/+*^ mice (Fig. 2j). These results indicate that TERT promotes enhanced stem cell competition independent of its catalytic activity.

Somatic deletion of *Tert* in mice with very long telomeres is unlikely to cause telomere dysfunction because achieving sufficient telomere shortening to cause telomere dysfunction requires generations of interbreeding for more than one year^16,31,32^. Critical telomere shortening in mouse and human cells triggers a DNA damage response and the activation of the p53 tumour suppressor protein, inducing either cell death or senescence^33^. To understand whether the impaired clone formation in TERT-deleted SSCs is mediated by p53, we generated *Trp53*^*flox/flox*^*:Tert*^*CreER/flox*^*:Rosa*^*lsl-Tdtomato/+*^ mice and examined competitive clone formation (Extended Data Fig. 4d). At day five, the homozygous deletion efficiency of the *Trp53-flox* alleles in tdTomato^+^ US was 44.4% (Extended Data Fig. 4e,f). We found that deleting p53 did not rescue the impaired clone formation associated with *Tert* inactivation, as measured by patch number, patch length and total patch length (Extended Data Fig. 4g–j). Consistent with these findings, we found no accumulation of γH2AX—a marker of DNA damage—in *Tert*^*CreER/flox*^ mice, whereas γH2AX was increased in G6 *Tert*^*Tdtomato/Tdtomato*^ mice with dysfunctional telomeres (Extended Data Fig. 4k,l). Together, these results show that the effect of TERT in promoting stem-cell-derived clone formation is independent of the canonical telomerase complex, catalytic activity and the DNA damage response.

## Differentiation of US cells lacking TERT

**Extended Data Fig. 5:**
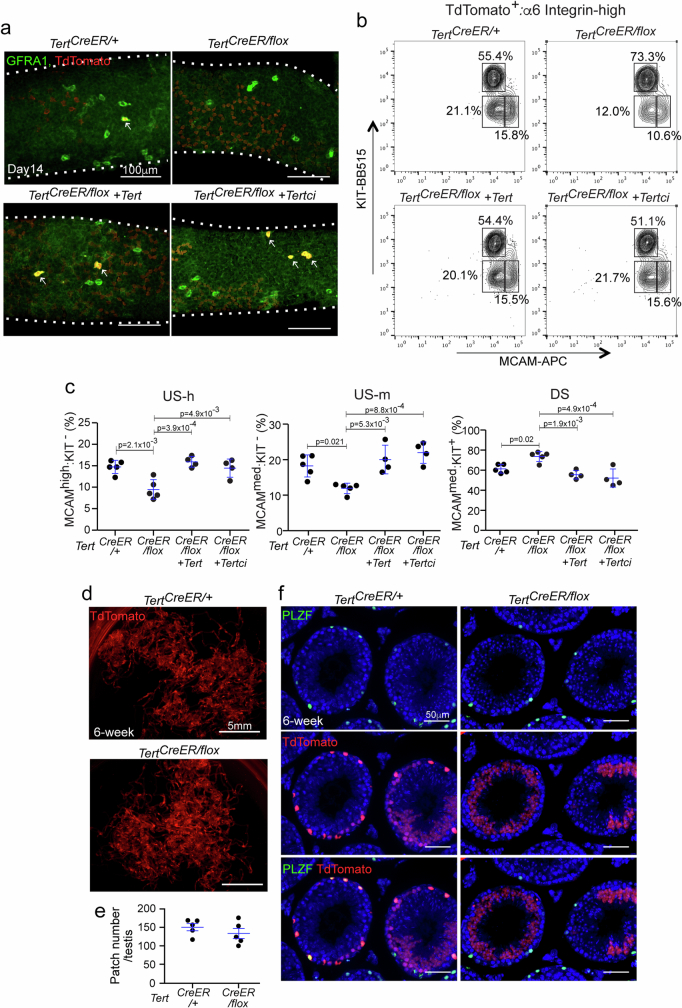
Differentiation in TERT-deleted US. **a**, Immunofluorescence for GFRA1 and tdTomato at 14 days post labelling. Scale bars, 100 μm. **b**, Flow cytometry analysis of testicular cells stained with α6-Integrin, MCAM, and KIT. tdTomato^+^, α6-Integrin-high cells were further separated by KIT and MCAM expression. **c**, Quantification of US-h, US-m, and DS populations (n = 5, 5, 4, 4 mice per group). *P* values were determined by one-way ANOVA with Tukey’s test. **d**, Epifluorescence for tdTomato in untangled seminiferous tubules at 6 weeks after 1 mg tamoxifen injection. Scale bars, 5 mm. **e**, Mean patch number at 6 weeks after tamoxifen injection (n = 5 mice per group). **f**, Immunofluorescence for tdTomato and PLZF using testis cross-sections at 6 weeks after tamoxifen injection. Scale bars, 50 μm. Data are represented as mean ± s.e.m.

**Extended Data Fig. 6:**
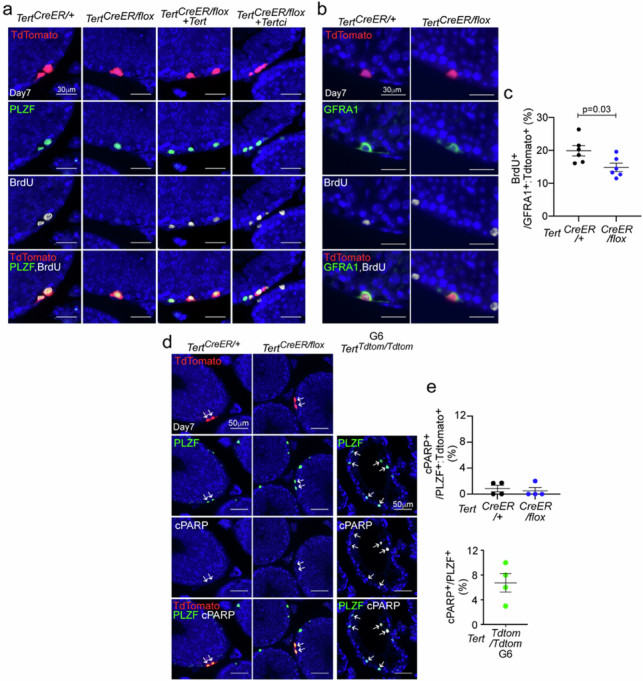
Proliferation and apoptosis in TERT-deleted US. **a**, Immunofluorescence for tdTomato, PLZF, and BrdU using testis cross-sections at 7 days after labelling. Scale bars, 30 μm. **b**, Immunofluorescence for tdTomato, GFRA1, and BrdU using testis cross-sections at 7 days after labelling. Scale bars, 30 μm. **c**, Quantification of GFRA1^+^ tdTomato^+^ BrdU^+^ US (n = 6 mice per group). *P* values determined by two-sided unpaired *t*-test. Data are represented as mean ± s.e.m**. d**, Immunofluorescence for PLZF, tdTomato, and apoptosis marker, cleaved-PARP (cPARP), on testicular cross-sections at 7 days after labelling. G6 *Tert*^*Tdtomato/Tdtomato*^ mice were used as positive control for cleaved-PARP staining. Scale bars, 50 μm. **e**, Quantification of PLZF^+^ tdTomato^+^ cleaved-PARP^+^ US (n = 4 mice per group). Data are represented as mean ± s.e.m.

The preferential elimination of conditionally TERT-deleted SSCs could be caused by a promotion of differentiation, impaired proliferation or increased apoptosis. To distinguish among these possibilities, we investigated how clonal deletion of TERT influences SSC fates across time points. At 14 days after tamoxifen administration, whole-mount immunofluorescence showed that tdTomato^+^ A_s_ and A_pr_ US were significantly decreased in *Tert*^*CreER/flox*^ mice and were rescued by TERT or TERTci (Fig. 2k and Extended Data Fig. 5a). Flow cytometry analysis revealed that tdTomato^+^ US-h and US-m cells were significantly decreased, whereas the KIT^+^ DS population was reciprocally increased, and the alterations in both the US and the DS populations were rescued by transgenic *Tert* or *Tertci* (Extended Data Fig. 5b,c). At six weeks, when a comparable frequency of labelled patches was found in *Tert*^*CreER/+*^ and *Tert*^*CreER/flox*^ mice, labelled clones contained significantly fewer PLZF^+^ US cells (Fig. 2l and Extended Data Fig. 5d–f). These results indicate that clonal TERT deletion promotes the differentiation of US cells to DS committed progenitor cells. To understand how the loss of TERT affects cell proliferation, we measured BrdU incorporation seven days after tamoxifen administration. The percentage of US cells incorporating BrdU was significantly reduced in *Tert*^*CreER/flox*^ mice, and proliferation was rescued with transgenic expression of *Tert* or *Tertci* (Fig. 2m and Extended Data Fig. 6a). The reduction of BrdU incorporation was also observed in GFRA1^+^ short-chain US (Extended Data Fig. 6b,c). There was no increase in apoptosis by cleaved-PARP staining in *Tert*^*CreER/flox*^ mice, but apoptosis was increased in testes from control G6 *Tert*^*Tdtomato/Tdtomato*^ mice with critically short telomeres (Extended Data Fig. 6d,e). Together, these data show that the impaired clone formation that is seen in TERT-deleted SSCs occurs because the loss of TERT promotes differentiation and decreased proliferation.

## Reduced chromatin accessibility in US cells

**Fig. 3:**
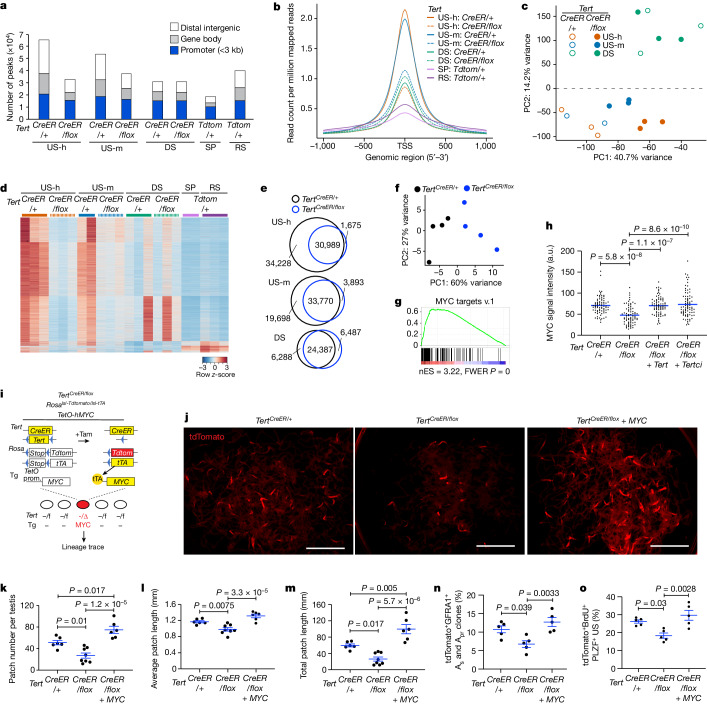
TERT deletion compromises chromatin accessibility and MYC pathway in SSCs. **a**, Peak calls from ATAC-seq data. Peak calls from each cell type are shown individually. Colour indicates the type of genomic region overlapped by the peak. **b**, Average tag density of ATAC-seq reads around TSSs. **c**, PCA of ATAC-seq data from US-h, US-m and DS cells purified from *Tert*^*CreER/+*^ versus *Tert*^*CreER/flox*^ mice. **d**, Heat map representation of 11,656 peaks that are significantly different between *Tert*^*CreER/+*^ and *Tert*^*CreER/flox*^ in US-h, US-m and DS or significantly more open either in SP or RS. Each row represents one ATAC-seq peak. Colour represents the relative ATAC-seq accessibility. **e**, Venn diagrams of the peaks in US-h, US-m and DS cells from *Tert*^*CreER/+*^ and *Tert*^*CreER/flox*^ mice. **f**, PCA of RNA-seq data from tdTomato^+^ US-h. **g**, Significant downregulation of MYC target genes in *Tert*^*CreER/flox*^ US-h cells. RNA-seq data were analysed using GSEA. FWER, family-wise error rate; nES, normalized enrichment score. **h**, Quantification of mean signal intensity for MYC staining in tdTomato^+^PLZF^+^ US using testicular cross-sections at seven days after labelling (*n* = 77 cells from 4 mice, 76 cells from 6 mice, 77 cells from 4 mice, 81 cells from 4 mice, left to right). *P* values determined by one-way ANOVA with Tukey’s test. a.u., arbitrary units. **i**, *Tert*^*CreER/flox*^*:Rosa*^*lsl-Tdtomato/lsl-tTA*^*:TetO-hMYC* mice. **j**, Epifluorescence for tdTomato in untangled seminiferous tubules at three months after labelling. Scale bars, 5 mm. **k**–**m**, Quantification of mean patch number (**k**), mean patch length (**l**) and total patch length (**m**) (*n* = 6, 8, 6 mice from left to right). *P* values determined by one-way ANOVA with Tukey’s test. **n**, Quantification of tdTomato^+^GFRA1^+^ A_s_ and A_pr_ clones at 14 days after labelling, detected by whole-mount immunofluorescence (*n* = 5 mice per group). *P* values determined by one-way ANOVA with Tukey’s test. **o**, Quantitative analysis of tdTomato^+^BrdU^+^PLZF^+^ US using testicular cross-sections at seven days after labelling (*n* = 5 mice per group). *P* values determined by one-way ANOVA with Tukey’s test. Data are mean ± s.e.m.

**Extended Data Fig. 7:**
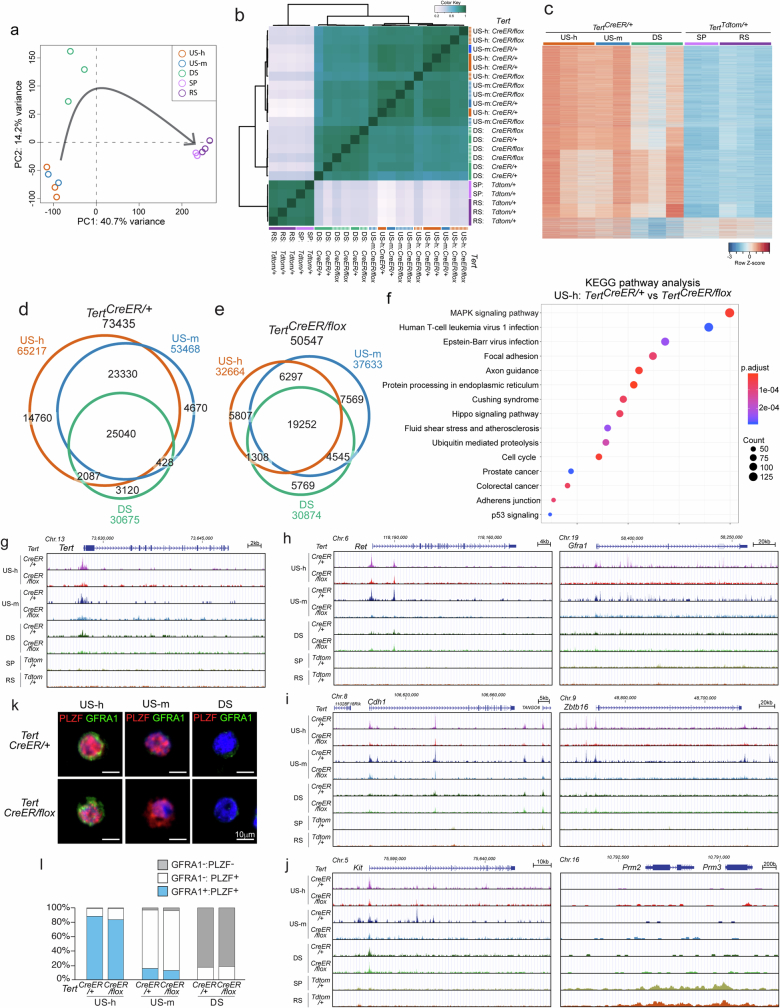
Global changes of open chromatin structure in US after acute deletion of TERT. **a**, PCA of ATAC-seq data. Colours represent indicated cell types from *Tert*-heterozygous mice. Arrow indicates direction of differentiation. **b**, Pearson correlation heat maps of ATAC-seq samples. **c**, Heat map representation of 7597 peaks showing significant differences across US-h, US-m, DS, SP, and RS. Each row represents one ATAC-seq peak. Colour represents relative ATAC-seq accessibility. **d**,**e**, Venn diagrams showing the number of peaks found in spermatogonia subpopulations (*Tert*^*CreER/+*^ (**d**) and *Tert*^*CreER/flox*^ (**e**)). **f**, KEGG pathway analysis of differential peaks between *Tert*^*CreER/+*^ and *Tert*^*CreER/flox*^ US-h. **g**–**j**, Read distribution of ATAC-seq data across different cell types. **g**, *Tert* locus. **h**, *Ret* and *Gfra1* loci. These genes are dominantly expressed in US-h. **i**, *Cdh1* and *Zbtb16* loci. These genes are dominantly expressed in both US-h and US-m. **j**, *Kit* and *Prm1/Prm2* loci. *Kit* is dominantly expressed in DS and *Prm1/Prm2* are dominantly expressed in RS. **k**, Immunofluorescence for GFRA1 and PLZF in sorted US-h, US-m and DS. **l**, Quantification of GFRA1^+^ and PLZF^+^ expression in sorted cells (n = 132, 154, 201, 125, 160, 180 cells from 3 mice per group from left to right).

To understand how TERT deletion affects global chromatin structure, we performed the assay for transposase-accessible chromatin with sequencing (ATAC-seq), which allows chromatin accessibility genome-wide to be assessed^34^. To first define the patterns of chromatin changes during normal spermatogenesis, we purified US-h, US-m and DS from *Tert*^*CreER/+*^ mice that had been injected with tamoxifen seven days before isolation, and spermatocytes (SP) and round spermatids (RS) were purified on the basis of differential *Tert* promoter activity in *Tert*^*Tdtomato/+*^ mice^16^. Principal component analysis (PCA) revealed that US-h and US-m clustered together in the bottom left quadrant, consistent with their similar patterns of gene expression^26^ (Extended Data Fig. 7a). DS cells localized in the top left quadrant, whereas SP and RS populations were clustered together in the bottom right quadrant (Extended Data Fig. 7a), indicating that the PC1 axis captures the changes in global chromatin state associated with differentiation. Similarly, Pearson correlation hierarchical clustering showed a high correlation in open chromatin patterns among spermatogonia subpopulations, but abrupt changes of chromatin accessibility globally after entry to meiosis (Extended Data Fig. 7b). The number of unique ATAC-seq peaks and promoter chromatin accessibility surrounding transcription start sites (TSSs) were highest in US-h and US-m and decreased significantly during differentiation into DS and SP (Fig. 3a,b and Extended Data Fig. 7c,d). The promoter region of *Tert* was accessible in US-h and US-m cells, less accessible in DS cells and inaccessible in SP and RS, consistent with the expression pattern of TERT during spermatogenesis^16^ (Extended Data Fig. 7g). Together, these data reveal that the population of US cells exhibits a markedly increased pattern of chromatin accessibility, and that a global reduction in chromatin accessibility occurs during lineage differentiation.

To understand how clonal TERT deletion influences chromatin accessibility, we performed ATAC-seq on US-h, US-m and DS isolated from *Tert*^*CreER/flox*^ mice and controls. The overall pattern of chromatin accessibility in TERT-deleted cells remained similar to that of *Tert*^*CreER/+*^ controls, on the basis of Pearson correlation hierarchical clustering (Extended Data Fig. 7b). However, PCA revealed that TERT deletion caused a shift in the US-h and US-m along the differentiation axis, whereas the loss of TERT had no discernible effect on committed DS (Fig. 3c). Deletion of TERT in the purified populations of US caused a marked reduction in the number of open chromatin peaks, and diminished chromatin accessibility surrounding TSSs to a level resembling that of the control DS (Fig. 3a,b,d,e and Extended Data Fig. 7e). By contrast, peak number was unaffected by TERT deletion in DS (Fig. 3a,b,d,e and Extended Data Fig. 7e). Pathway analysis revealed that genes in the MAPK signalling pathway, which promotes self-renewal of SSCs^35^, were particularly enriched among those showing a loss of open chromatin peaks in the TERT-deleted US (Extended Data Fig. 7f). The reduction in open chromatin peaks was also evident in genes associated with stemness in SSCs, including *Ret*, *Gfra1*, *Cdh1* and *Zbtb16*, whereas those associated with differentiation, including *Kit*, *Prm2* and *Prm3*, remained unchanged (Extended Data Fig. 7h–j). Immunostaining revealed similar levels of GFRA1 and PLZF expression between sorted US-h, US-m and DS from *Tert*^*CreER/+*^ and *Tert*^*CreER/flox*^ mice, suggesting that the alteration of global chromatin accessibility is not due to changes in the composition of sorted cells (Extended Data Fig. 7k,l). Together, these data show that clonal inactivation of TERT causes a loss of open chromatin selectively in the stem-cell-containing population, but not in committed progenitors, consistent with a model of enhanced stem cell differentiation caused by TERT deletion.

## Rescue of clone formation with MYC

**Extended Data Fig. 8:**
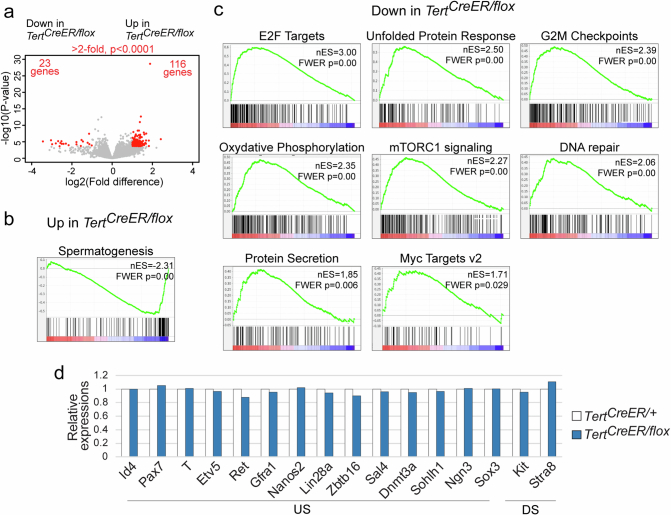
RNA-seq analysis of TERT-deleted US-h. **a**, Volcano plot of RNA-seq data using purified tdTomato^+^ US-h from *Tert*^*CreER/+*^ and *Tert*^*CreER/flox*^ mice. Genes showing the significant changes (more than twofold change and *P* < 0.0001) are coloured in red. **b**, Gene set up-regulated in US-h purified from *Tert*^*CreER/flox*^ mice. **c**, Gene sets down-regulated in US-h purified from *Tert*^*CreER/flox*^ mice. **d**, Gene expression of US and DS marker genes in RNA-seq data.

**Extended Data Fig. 9:**
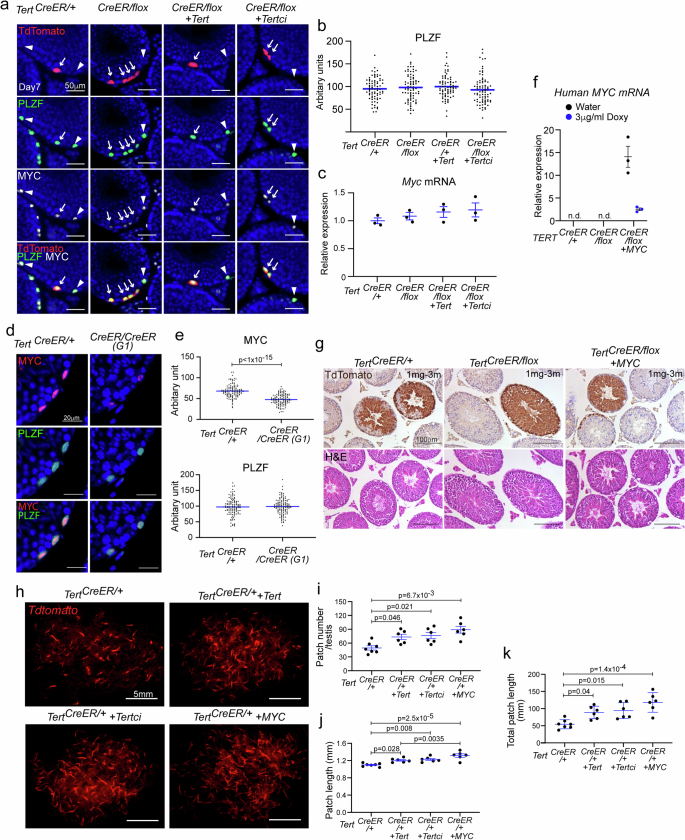
Downregulation of MYC in TERT-deleted US. **a**, Immunofluorescence for MYC, PLZF, and tdTomato at day 7 after tamoxifen labelling. Scale bars, 50 μm. **b**, Quantification of mean signal intensities for PLZF staining in US (n = 77 cells from 4 mice, 76 cells from 6 mice, 77 cells from 4 mice, 81 cells from 4 mice from left to right). **c**, qRT–PCR analysis of mouse *Myc* mRNA in purified tdTomato^+^ US at day 7. Doxycycline was added to drinking water. Expression was normalized with *Actb* (n = 3 mice per group). **d**, Immunofluorescence for MYC and PLZF in testicular cross-sections. Scale bars, 20 μm. **e**, Quantification of mean signal intensities for MYC and PLZF staining in US (n = 115, 117 cells from 4 mice per group from left to right). *P* values determined by two-sided unpaired *t*-test. **f**, qRT–PCR analysis of transgenic human *MYC* mRNA in purified tdTomato^+^ US at day 7 after tamoxifen labelling. The primers and probe used for this assay were human-specific and do not detect mouse *Myc*. Doxycycline was added to drinking water. Expression was normalized with *Actb* (n = 3 mice per group). n.d.; not detected. **g**, Immunostaining against tdTomato and histology of testes at 3-month after labelling. Scale bars, 100 μm. **h**, Epifluorescence for tdTomato in untangled seminiferous tubules at 3-month after 1 mg tamoxifen administration. Scale bars, 5 mm. **i**–**k**, Quantitative data of patch number (**i**), average patch length (**j**) and total patch length (**k**) (*n* = 7, 6, 6, 6 mice from left to right). *P* values determined by one-way ANOVA with Tukey’s test. Data are represented as mean ± s.e.m.

To understand how TERT promotes competitive clone formation in SSCs, we examined gene expression in TERT-deleted US-h cells by RNA sequencing (RNA-seq) seven days after tamoxifen administration. PCA showed that TERT-deleted US-h clustered separately from TERT^+^ controls (Fig. 3f). Using strict cut-offs for significance, there were 23 genes downregulated and 116 genes upregulated in TERT-deleted US-h (Extended Data Fig. 8a and Supplementary Table 1). Gene set enrichment analysis (GSEA) revealed that spermatogenesis-related genes were upregulated in TERT-deleted US-h, consistent with enhanced differentiation (Extended Data Fig. 8b). Several gene sets were downregulated in TERT-deleted US-h cells, including E2F targets and G2M checkpoints, reflecting the quantitative reduction in proliferation in these cells (Extended Data Fig. 8c). The most significantly downregulated gene set was ‘MYC targets v.1’, and a second gene set, ‘MYC targets v.2’, was also represented (Fig. 3g and Extended Data Fig. 8c). These changes were not caused by alterations in the composition of sorted cells, because the expression levels of stem cell and differentiation marker genes remained unchanged (Extended Data Fig. 8d). Consistent with the GSEA, MYC protein levels were significantly decreased in TERT-deleted US by immunofluorescence analysis, and MYC levels were restored by transgenic expression of TERT or TERTci (Fig. 3h and Extended Data Fig. 9a,b). *Myc* mRNA levels remained unchanged, which suggests that TERT promotes MYC expression at the post-transcriptional level (Extended Data Fig. 9c). Notably, MYC protein was also reduced in US cells from germline *Tert*-knockout mice (*Tert*^*CreER/CreER*^ G1 mice) (Extended Data Fig. 9d,e), indicating that a reduction in MYC levels is linked to TERT deletion, and is not a feature of impaired cell competition.

MYC is a transcription factor that promotes cell competitiveness by regulating cell proliferation, growth and metabolism^20,36–38^. MYC has been shown to promote SSC self-renewal and is also an important oncogene^39–42^. Given the reduction in MYC seen with clonal TERT inactivation, we hypothesized that overexpressing MYC might rescue the failure of clone formation in TERT-deleted SSCs. To test this idea, we intercrossed *Tert*^*CreER/flox*^*:Rosa*^*lsl-Tdtomato/lsl-tTA*^ mice with *TetO-human MYC* transgenic mice (*Tert*^*CreER/flox*^ + *MYC*) (Fig. 3i). This system allows simultaneous deletion of the residual *Tert* allele and activation of transgenic MYC selectively in a lineage of TERT-expressing stem cells. To limit the expression levels of transgenic MYC, mice were treated with doxycycline, which reduced transgenic *MYC* mRNA levels by 5.6-fold (Extended Data Fig. 9f). Three months after tamoxifen treatment, the defects in clone formation associated with TERT loss were considerably rescued by MYC expression, as measured by patch number, patch length and total patch length (Fig. 3j–m). MYC overexpression did not impair spermatogenesis (Extended Data Fig. 9g). MYC expression also restored the number of GFRA1^+^ cells and proliferation of TERT-deleted US (Fig. 3n,o). To examine the effects of overexpression of TERT, TERTci and MYC in SSC competition, we induced these transgenes in a TERT-competent *Tert*^*CreER/+*^ context. At three months after labelling, patch number, patch length and total patch number were increased (Extended Data Fig. 9h–k), indicating that overexpression of these transgenes is sufficient to enhance SSC competition. Although we did not find defects in spermatogenesis, overexpression of these transgenes might result in non-physiological effects. Together, these results indicate that TERT promotes stem cell competition through MYC, and establish an epistatic relationship between TERT and MYC.

## Mild telomere shortening with TERT loss

**Fig. 4:**
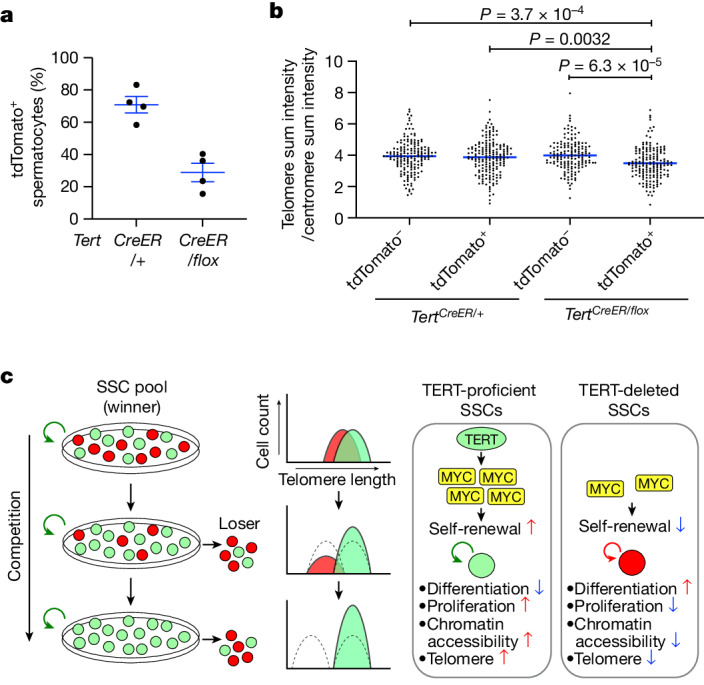
TERT-dependent cell competition eliminates SSCs with shorter telomeres. **a**, Quantification of tdTomato^+^ spermatocytes at one year after the administration of 5 mg tamoxifen (*n* = 4 mice per group). Data are mean ± s.e.m. **b**, Quantification of telomere signals in spermatocytes at one year after labelling (*n* = 168, 179, 155, 171 cells from 3 mice per group from left to right). Total telomere signals per cell (telomere sum signals) were normalized with total centromere signals per cell (centromere sum signals). *P* values determined by one-way ANOVA with Tukey’s test. Data are mean values. **c**, Summary schematic of the functions of TERT in SSC competition. TERT-deleted SSCs are progressively eliminated from the SSC pool through cell competition, reducing the contribution of TERT-deleted SSCs that have shorter telomeres to spermatogenesis over time (left). Green and red indicate TERT^+^ and TERT-deleted cells, respectively. In wild-type SSCs, TERT promotes competitive clone formation by upregulating MYC protein. Deletion of TERT in SSCs downregulates MYC, promotes differentiation, compromises proliferation, induces a global loss of chromatin accessibility and accelerates telomere shortening.

**Extended Data Fig. 10:**
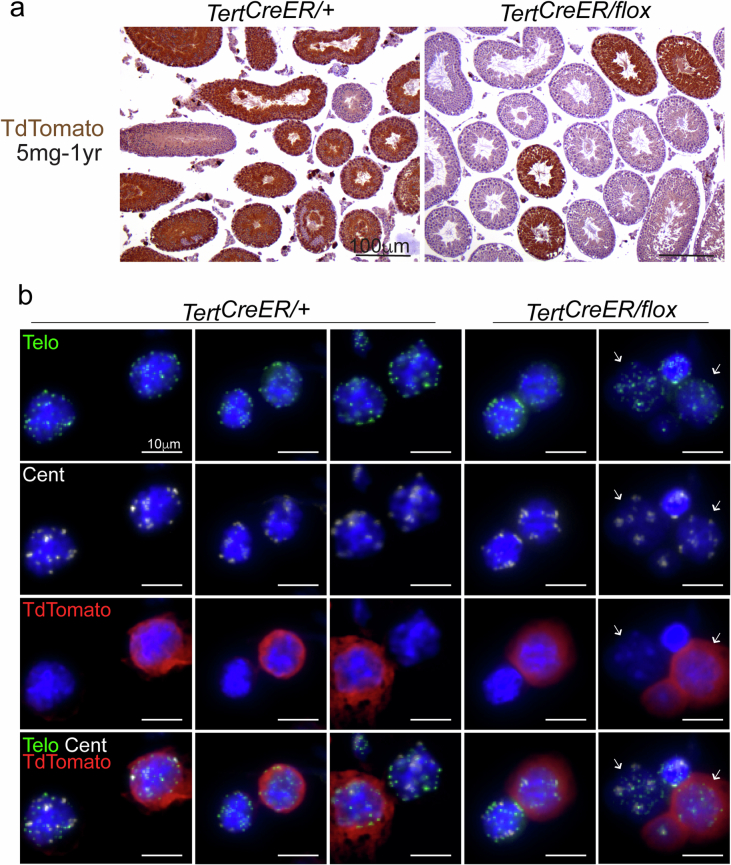
Telomere length in TERT-deleted male germ cells. **a**, Immunostaining for tdTomato using testes at one year after 5 mg tamoxifen injections. Scale bars, 100 μm. **b**, FISH for telomeres and centromeres and immunofluorescens for tdTomato using spermatocytes one year after 5 mg tamoxifen injections. Arrows in the right panels indicate spermatocytes. Scale bars, 10 μm.

The non-canonical effects of TERT in promoting stem cell competition by disfavouring clones that have lost TERT could affect tissue telomere lengths. To investigate this possibility, we measured relative telomere lengths in spermatocytes in *TERT*^*CreER/flox*^ mice and *TERT*^*CreER/+*^ controls. One year after the injection of tamoxifen at a high dose, tdTomato^+^ male germ cells were less abundant in *TERT*^*CreER/flox*^ mice compared with *TERT*^*CreER/+*^ controls by immunofluorescence of single cells and by immunohistochemistry on tissue sections (Fig. 4a and Extended Data Fig. 10a). Single-cell suspensions from the testes were analysed by combined fluorescence in situ hybridization (FISH) for telomeres and centromeres and by immunofluorescence for tdTomato. In tdTomato^+^ spermatocytes from *TERT*^*CreER/flox*^ mice, telomere length was reduced by 12.7% compared with tdTomato^−^ spermatocytes, owing to the loss of TERT (Fig. 4b and Extended Data Fig. 10b). For comparison, telomere lengths were indistinguishable in tdTomato^+^ and tdTomato^−^ spermatocytes from *TERT*^*CreER/+*^ controls. These findings show that the non-canonical effects of TERT in enhancing stem cell competition can serve to cull cells that have lost TERT and that suffer only modest telomere shortening (Fig. 4c). These observations provide a new mechanism for favouring cells with long telomeres to ensure that telomeres are maintained in sperm and in the early embryo.

## Discussion

Telomerase serves essential functions in tissue stem cells in maintaining telomeres, although the effects of telomerase loss become evident after an extended lag phase during which telomeres progressively shorten^12,13,15^. By tracing the fate of individual SSCs in vivo, we found that inactivating TERT in SSCs promotes rapid stem cell differentiation, global loss of chromatin accessibility, reduced MYC expression and impaired clone formation, as these TERT-deleted stem cells are outcompeted by unlabelled SSCs. This non-canonical effect of TERT in facilitating stem cell competition is independent of catalytic function and occurs without a notable lag phase—a fundamental feature of senescence and crisis enforced by critically short telomeres. Our results lead us to propose a model in which the elimination of clones derived from TERT-deleted SSCs represents a means of culling cells with only modest telomere shortening from the population of sperm, and can therefore serve to maintain telomere lengths during life in the testes, as lineages deriving from SSCs with the highest TERT levels outcompete those deriving from SSCs with lower levels of TERT (Fig. 4c). Although the checkpoint responses to telomere dysfunction effectively eliminate cells with very short telomeres, the non-canonical mechanisms identified here provide a means of removing cells with only modest telomere shortening, long before telomere dysfunction ensues. Our findings establish TERT as a key determinant regulating competition between tissue stem cells by favouring self-renewal and disfavouring differentiation. This result is noteworthy in that germline inactivation of *Tert* in mice is well tolerated while telomeres are sufficiently long. The apparent absence of stem cell defects in telomerase-knockout mice with long telomeres might occur because of genetic buffering or compensation, or because of a lack of competition in a tissue in which all cells lack TERT. In this context, germline gene inactivation identifies only one layer of activity, whereas clonal, conditional gene deletion can reveal deeper aspects of gene function.

Although TERT levels are important in the clonal expansion of stem cells, they might have a similar role during pre-neoplastic processes and during carcinogenesis. Germline *TERT* polymorphisms confer an increased risk of clonal haematopoiesis and an increased susceptibility to diverse cancer types^43,44^. It is notable that somatic TERT promoter mutations that activate TERT transcription show strong positive selection during tumour progression, as the prevalence of these mutations increases substantially from pre-invasive to invasive stages. Our data showing that TERT-deleted SSCs exhibit reduced levels of MYC provide support for this model. MYC activity is central in determining outcomes in cell competition: cells expressing higher levels of MYC outcompete those with lower MYC levels, and MYC represents a key node in many human cancers^20,36–38^. Thus, upregulation of TERT might promote the clonal expansion of cancer cells by activating the MYC pathway, in addition to maintaining telomere function. Together, these data establish a non-canonical role for TERT in promoting clonal competition in stem cells in vivo, with implications for understanding tissue homeostasis, cancer development and telomere maintenance.

## Methods

### Animals

All animal experiments were approved by the Administrative Panel on Laboratory Animal Care (APLAC) in protocol APLAC-12684, and all experiments were in compliance with the ethical regulations of Stanford University. Mice were housed at an ambient temperature of 22 °C and at 40% humidity, with a 12-h light–dark cycle (07:00–19:00). *Tert-CreER*, *TetO-Tert*, *TetO-Tertci* and *Tert-Tdtomato* mice were previously reported^3,16,27^. *Rosa-lslTdtomato*^45^, *Rosa-lsl-tTA*^46^, *TetO-hMYC*^47^, *Terc-KO*^48^ and *Trp53-flox*^49^ mice were purchased from The Jackson Laboratory. Tamoxifen (Cayman) was dissolved in corn oil (Sigma-Aldrich) at 5–20 mg ml^−1^ by incubating at 50 °C for 30 min with mixing every 5 min. Two- to four-month-old mice were administered with 0.25–4 mg tamoxifen per 25 g body weight by oral gavage or intra-peritoneal injection. Doxycycline (Sigma-Aldrich) was dissolved in drinking water in light-protected bottles at 1 or 3 μg ml^−1^ and changed every three or four days. BrdU (Sigma-Aldrich) was dissolved in PBS at 10 mg ml^−1^ and intraperitoneally injected at 1.25 mg per 25 g body weight two hours before euthanasia.

### Generation of *Tert-flox* mice

A 9-kb fragment of the *Tert* locus was subcloned and a Lox-Puro-lox cassette from the pBS.DAT-LoxStop plasmid (a gift from D. Tuveson) was inserted at the BsiWI site in the second intron. Another loxP sequence and NdeI site were inserted at the KasI site in the sixth intron. The targeting vector was linearized and electroporated into J1 mouse ES cells. After positive selection with puromycin, correctly targeted ES clones were selected by long-range PCR and Southern blotting, and then injected into C57BL6 blastocysts to generate the knock-in line. To remove floxed puro cassette, the knock-in line was crossed with CMV-cre mice^50^ and puro-negative *Tert*-floxed mice were selected by PCR and Southern blotting using genomic DNA from tail tips. *TERT*^*flox/+*^ mice were born at normal Mendelian frequency. Original uncropped images of Southern blots are provided in Supplementary Fig. 1.

### Lineage-tracing assay

After tamoxifen injection, testes were detunicated and seminiferous tubules were untangled using fine forceps in PBS containing 1 mg ml^−1^ collagenase IV (Worthington) for 10 min, and placed in cold PBS. Images were captured with a fluorescent dissection microscope and the patch number and length were measured with ImageJ. Total patch length was calculated by multiplying the patch number by the average patch length.

### Sperm count

Cauda epididymides were dissected into small pieces and incubated in potassium simplex optimized medium (KSOM) at 37 °C for one hour under 5% CO_2_ to allow sperm to exude. The collected sperm were then fixed with 4% PFA and counted with a haemacytometer.

### Whole-mount immunofluorescence of seminiferous tubules

Seminiferous tubules were dissociated using fine forceps in PBS containing 1 mg ml^−1^ collagenase IV for 10 min, fixed with 4% PFA at 4 °C for two hours, cleared with 0.1% Igepal CA-630 (Sigma-Aldrich) in PBST and dehydrated and rehydrated by immersing in a gradient of methanol diluted with PBST (25%, 50%, 75%, 100%, 75%, 50%, 25%) at 4 °C for 5 min each. After washing in PBST, tubules were incubated in blocking buffer (0.5% BSA in PBST), followed by incubation with antibodies in Immuno Shot Immunostaining, Mild (Cosmo Bio) at 4 °C for two days. After extensive washing with PBST, tubules were incubated with secondary antibodies in blocking buffer at room temperature for 90 min, washed with PBST and then mounted in Vectashield with DAPI (Vector Laboratories). Images were captured on a Leica SP5 confocal microscope and processed in Photoshop CC or later versions. Syncytium of US (A_s_–A_16_) were visually judged on the basis of continuous E-cadherin, GFRA1 or tdTomato staining. The following antibodies were used: anti-RFP (Abcam, ab124754, rabbit polyclonal, 1:500 dilution); anti-RFP (MBL, M208-3, mouse monoclonal 1G9 and 3G5, 1:200 dilution); anti-E-cadherin (R&D systems, AF748, goat polyclonal, 1:200 dilution); anti-GFRA1 (R&D systems, AF560, goat polyclonal, 1:200 dilution); and anti-KIT (Cell Signaling Technology, 3074, rabbit monoclonal D13A2, 1:200 dilution).

### Section immunostaining

Testes were detunicated, fixed with 4% PFA at 4 °C overnight, incubated in a gradient of ethanol and xylen, embedded in paraffin and cut into 5-μm sections. After rehydration, antigen retrieval was performed using either citric acid or tris-based antigen retrieval solution (Vector Laboratories) for 5 min in a pressure cooker. Sections were blocked with 0.5% BSA in PBST and incubated with primary antibody at 4 °C overnight. After washing with PBST, sections were incubated with secondary antibodies at room temperature for 1 h and mounted in Vectashield with DAPI. For BrdU detection, slides were treated with 2 M HCl for 20 min, blocked with 0.5% BSA in PBST and incubated with rabbit anti-PLZF and rat anti-BrdU antibodies at 4 °C overnight, and signals were detected by Alexa 488-conjugated anti-rat IgG and Cy5-conjugated anti-rabbit IgG antibodies. For co-staining using rabbit anti-RFP antibodies and rabbit anti-PLZF antibodies, sections were antigen retrieved, blocked with 0.5% BSA in PBST, incubated with anti-RFP antibody, then with HRP-conjugated anti-rabbit secondary antibody as described above, and signals were detected with the TSA Plus Cyanine 3 system (Akoya Biosciences). After signal detection, the antibodies were stripped off by antigen retrieval, and sections were further stained with other antibodies. For triple staining using rabbit anti-RFP, rabbit anti-PLZF and rabbit anti-MYC antibodies, sections were stained with anti-RFP antibody using the TSA Plus Cyanine 3 system, and antibodies were stripped off with antigen retrieval. Then, those sections were stained with anti-MYC antibody with the TSA Plus Fluorescein system (Akoya Biosciences), followed by antigen retrieval to remove antibodies. Finally, the sections were further stained with anti-PLZF antibody and Cy5-conjugated anti-rabbit IgG. Slides were mounted in Vectashield with DAPI. For chromogenic staining for tdTomato, sections were incubated with anti-RFP antibody followed by HRP-conjugated antiboy. Signals were detected with a DAB substrate kit (Vector Laboratories). Sections were counterstained with haematoxylin, dehydrated with ethanol and xylene and then mounted in Clearmount (American MasterTech). To quantify PLZF^+^ cells, seminiferous tubules in stage VII–VIII were excluded from the analyses to prevent the inclusion of PLZF^+^ early DS cells. Images were captured on a fluorescent microscope and processed in Photoshop. The signal intensities of MYC and PLZF were quantified with ImageJ. Immunofluorescence data were captured using Leica Application Suite AF and immunohistochemistry data were captured using Leica LAS 4.2. The following antibodies were used: anti-RFP (Abcam, ab124754, rabbit polyclonal, 1:500 dilution for immunofluorescence without TSA or 1:3,000 dilution for immunofluorescence with TSA); anti-BrdU (Bio-Rad, MCA2483, mouse monoclonal Bu201, 1:500 dilution); anti-GFRA1 (R&D systems, AF560, goat polyclonal, 1:200 dilution); anti-PLZF (Santa Cruz, sc-22839, rabbit monoclonal H-300, 1:200 dilution for immunofluorescence without TSA or 1:5,000 dilution for immunofluorescence with TSA); anti-cleaved PARP (Cell Signaling Technology, 9548, mouse monoclonal 7C9, 1:500 dilution); anti-γH2AX (EMD Millipore, 05-636, mouse monoclonal JBW301, 1:2,000 dilution); and anti-MYC (Cell Signaling Technology, 13987, rabbit monoclonal D3N8F, 1:500 dilution).

### TRAP assays

A two-step TRAP (telomeric repeat amplification protocol) procedure was performed as previously reported^51^. Extracted fractions from whole testis at three weeks or fluorescence-activated cell sorting (FACS)-sorted US were incubated with telomeric primers for a 30-min initial extension step at 30 °C in a PCR machine, followed by 5 min inactivation at 72 °C. Without purification, 1 μl of the extended reaction was PCR amplified (cycles of 30 s at 94 °C, followed by 30 s at 59 °C) in the presence of ^32^P end-labelled telomeric primers that had been purified using a micro-spin G-25 column (GE Healthcare). PCR reactions were resolved by 9% polyacrylamide gel electrophoresis at room temperature, and the gel was exposed to a phosphor imager and scanned by a Typhoon scanner. The scanned image was quantified using the TotalLab Quant software. Representative gel images were presented among at least two repeats. Original uncropped images are provided in Supplementary Figs. 1 and 2.

### FACS analysis

Testes were detunicated, lightly dissociated in PBS and incubated in PBS containing 1 mM EDTA, 1 mg ml^−1^ collagenase I (Worthington) and DNase I (Worthington) at 32 °C for 8 min. Cells were centrifuged at 250*g* for 5 min and the supernatant was removed. After repeating the collagenase I treatment, testicular cells were further digested with TrypLE Express (Gibco) at 32 °C for 15 min. During enzymatic digestions, seminiferous tubules were mechanically fragmented with vigorous pipetting every 5 min. Cells were sequentially filtered with 70-μm and 40-μm strainers, resuspended in cold FACS buffer (2% FBS and 1 mM EDTA in PBS) and incubated with antibodies on ice for 30 min. After washing with PBS, cells were resuspended in cold FACS buffer containing DAPI, and analysed and sorted with a BD Aria II (BD Biosciences). Flow cytometry data were acquired using BD FACSDiva software v.8.0. Data were analysed with FlowJo v.9 software. The following antibodies were used; anti-α6 integrin with Pe/Cy7 (Biolegend, 313622, rat monoclonal GoH3, 1:150 dilution); anti-MCAM with APC (Biolegend, 134712, rat monoclonal ME-9F1, 1:200 dilution); and anti-KIT with BB515 (BD Biosciences, 564481, mouse monoclonal 2B8, 1:200 dilution). The gating strategy is provided in Supplementary Fig. 3.

### Telomere FISH

For preparing cytospin slides of testicular cells, single-cell suspensions were prepared as described above and resuspended in PBS. Cells were then fixed with 4% PFA at room temperature for 10 min. After washing, cells were resuspended in PBS and cytospun at 250*g* for 5 min. Slides were stored in 70% ethanol at 4 °C. For telomere FISH combined with antibody staining against tdTomato, slides were hydrated in PBS, blocked with PBST containing 0.5% BSA and then incubated with anti-RFP antibody at 4 °C overnight. After washing with PBST, slides were incubated with HRP-conjugated anti-rabbit secondary antibody at room temperature for 30 min and washed with PBST. Signals were detected using the TSA Plus Cyanine 3 system. For telomere FISH, slides after antibody staining were washed with PBS, and dehydrated by immersing in 70%, 90% and 100% ethanol. After drying, slides were incubated in hybridization buffer (70% formamide, 10 mM Tris-HCl pH 7.4 and 0.5% blocking reagent (Roche)) containing 0.2 μM Alexa 488-conjugated telomere probes (PNA Bio) and 0.2 μM Cy5-conjugated centromere probes (PNA Bio) at 80 °C for 10 min and then at 4 °C overnight. Slides were washed with 70% formamide containing 10 mM Tris-HCl pH 7.4 for 15 min twice, then with PBS, and were then mounted in Vectashield with DAPI. Images were captured on a fluorescent microscope and processed in Photoshop. Telomere sum signals and centromere sum signals were determined using Telometer v.3.0.5. Anti-RFP antibody (Abcam, ab124754, rabbit polyclonal, 1:500 dilution) was used.

### RNA in situ hybridization

At five days after labelling, testes were collected and tdTomato^+^ US were FACS-sorted, and cytospun at 200*g* for 5 min onto slides. Slides were fixed in 4% PFA for 30 min at room temperature and processed for single-molecule RNA FISH using the RNAscope 2.5 HD Reagent KIT—RED (Advanced Cell Diagnostics) and probes against mouse *Tert* or *Trp53* (Advanced Cell Diagnostics) according to the manufacturer’s instructions.

### qRT–PCR

For qRT–PCR, cells were directly sorted into Trizol LS (Thermo Fisher Scientific) by FACS. RNA was purified using the Direct-zol RNA Microprep kit (Zymo Research) and cDNA was synthesized using oligo-dT and the SuperScript IV First-Strand Synthesis system (Thermo Fisher Scientific). For qRT–PCR of *Tert* exon 2, *Tert* exon 6 and mouse *Myc*, TaqMan Fast Advanced Master Mix (Thermo Fisher Scientific) was used, along with Universal Probe Library Probes: no. 66 for *Tert* exon 2, no. 93 for *Tert* exon 6 and no. 77 for mouse *Myc* (Roche). For other qRT–PCR, the PowerUp SYBR Green Master Mix (Thermo Fisher Scientific) was used according to the product manual. PCR analysis was done with a 7900HT Fast Real-Time PCR System machine (ABI). Primer information is available in Supplementary Table 2.

### RNA-seq

US-h cells were directly sorted into Trizol LS by FACS and RNA was purified using the Direct-zol RNA Microprep kit. Genomic DNA was digested with on-column DNase treatment. RNA quality was checked by Bioanalyzer 2100 (Agilent). RNA-seq libraries were constructed using the SMARTer Stranded Total RNA-seq Kit v2—Pico Input Mammalian (Clontech), starting from 5 ng total RNA. cDNA was synthesized and amplified according to the manual. After the rRNA removal step, cDNA was amplified with 13 cycles of PCR reactions. The quality of purified cDNA libraries was confirmed by Bioanalyzer 2100. Libraries were sequenced on the Illumina NextSeq platform, generating about 16 million–24 million 75-bp paired-end reads per library. Four biological replicates per sample were analysed. Raw reads were trimmed by TrimGalore v.0.4.0 (Babraham Bioinformatics), mapped to mm10 by TopHat v.2.0.13 and analysed by DESeq2.

### ATAC-seq

ATAC-seq libraries were made as described previously^52^ using the Omni-ATAC protocol. Adjustments to the protocol were made to reflect two main features of the cell types profiled in this work. First, the amount of Tn5 transposase added to each reaction was modulated to maintain proportionality with the number of cells assayed. For example, a normal reaction uses 50,000 cells and 2.5 μl of Tn5 transposase in a 50-μl reaction. In the case of rarer SSCs, only 5,000 cells could be obtained so only 0.25 μl of Tn5 transposase was used in a 50-μl reaction. The difference in volume was adjusted using water. Second, the ploidy of each cell type was taken into account and the amount of Tn5 was adjusted on the basis of ploidy as well. For example, round spermatid cells are haploid, so the transposition of 50,000 cells would require 1.25 μl of Tn5 transposase in a 50-μl reaction. Similarly, spermatocytes are 4N meiotic cells so the amount of Tn5 transposase was increased proportionately and the amount of water in the reaction was reduced. In all cases, regardless of cell number or ploidy, the reaction volume of the transposition reaction was kept constant at 50 μl. All ATAC-seq reactions were performed using homemade Tn5 transposase and Tagment DNA buffer^53^. Downstream amplification and purification of libraries was performed as described previously^34,52,54^.

Preprocessing of ATAC-seq data was completed using the PEPATAC pipeline (https://pepatac.databio.org/). The mm10 genome build (https://github.com/databio/refgenie) was used for alignment. In brief, all fastq files were first trimmed to remove the Illumina Nextera adapter sequence using Skewer with “-f sanger –t 20 –m pe –x” options. FastQC (https://www.bioinformatics.babraham.ac.uk/projects/fastqc/) was used to validate proper trimming and check overall sequence data quality. Bowtie2 was then used for pre-alignments to remove reads that would map to chrM (revised Cambridge Reference Sequence), alpha satellite repeats, Alu repeats, ribosomal DNA repeats and other repeat regions with “-k 1 -D 20 -R 3 -N 1 -L 20 -i S,1,0.50 -X 2000 --rg-id” options. Bowtie2 was then used to align to the mm10 reference genome using “--very-sensitive -X 2000 --rg-id” options. SAMtools was used to sort and isolate uniquely mapped reads using “-f 2 -q 10 -b -@ 20” options. Picard (http://broadinstitute.github.io/picard/) was used to remove duplicates. Then the bam files were merged by conditions, and MACS2 was used to call peaks with parameter “-q 0.05 --nomodel --shift 0”. The narrow peaks were then filtered by the ENCODE 7 hg19 blacklist, as well as peaks that extend beyond the ends of chromosomes. Bedtools was used to retrieve the reads of the called peaks for each sample with the multicov module. All of the samples have a similar sequencing depth, mitochondrial rate and duplication rate. The spermatocyte and the round spermatid samples have a similar sequencing depth compared with all other samples, but a slightly higher mitochondrial rate and lower duplication rate, so have more final reads after initial processing and filtering. To make all the samples comparable for the statistical analysis, we used final reads as the normalization factor. The R package DESeq2 was used for statistical analysis to identify significant peaks between different conditions. The differential peaks were called between US-h CreER/+ and US-h CreER/flox samples. Peaks with FDR < 0.01 and a fold change larger than 2 or smaller than −2 were considered significant. The R package ChIPseeker was used for peak annotation. The R package ngsplot was used for visualizing the cumulated peak signal.

### Statistics and reproducibility

No statistical methods were used to predetermine sample sizes. All of the experiments were replicated more than twice and were statistically analysed and presented in the paper. When comparing two groups, *P* values were determined by two-sided unpaired *t*-test. When comparing more than two groups, *P* values were determined by one-way ANOVA with Tukey’s test. Values are presented as mean ± s.e.m. The mice were randomly assigned to each experimental or control group. Statistics and plots were generated by ggplot2 in R and GraphPad Prism 8.

### Reporting summary

Further information on research design is available in the Nature Portfolio Reporting Summary linked to this article.

## Online content

Any methods, additional references, Nature Portfolio reporting summaries, source data, extended data, supplementary information, acknowledgements, peer review information; details of author contributions and competing interests; and statements of data and code availability are available at 10.1038/s41586-024-07700-w.

### Supplementary information

Supplementary Information Supplementary Tables 1 and 2 and Supplementary Figs. 1–3.

Reporting Summary

## Data Availability

All data that support the finding of this study are available in a publicly accessible repository. The source data for the RNA-seq and ATAC-seq study are available in the NCBI Gene Expression Omnibus repository under accession number GSE14659. The resulting fastq files were aligned to the mouse reference genome (mm10).
