# Supplementary material for: Clonal inactivation of TERT impairs stem cell competition
Source: Nature. 2024 Jul 17;632(8023):201–8. doi: 10.1038/s41586-024-07700-w (PMC11291281; doi:10.1038/s41586-024-07700-w)
Supplement: Supplementary file 1 — Supplementary Tables 1 and 2 and Supplementary Figs. 1–3. [file 41586_2024_7700_MOESM1_ESM.pdf]

nature portfolio

https://doi.org/10.1038/s41586-024-07700-w

Supplementary information

Clonal inactivation of TERT impairs stem cell competition

In the format provided by the authors and unedited

SI table1: Differentially expressed genes

Down-regulated in Tert-CreER/flox

baseMean	log2 Fold Change	lfcSE	stat	pvalue	padj	entrez	gene_symbol	ensembl	
118.612	1.148	0.208	5.508	0.000000036	0.000038578	22755	Zfp93	ENSMUSG00000055305	zinc finger protein 93
16.621	4.149	0.822	5.048	0.000000446	0.000220830	64435	Fcamr	ENSMUSG00000026415	Fc receptor, IgA, IgM, high affinity
13.076	3.971	0.812	4.889	0.000001014	0.000406308	71520	Grap	ENSMUSG00000004837	GRB2 related adaptor protein
130.857	1.524	0.323	4.717	0.000002392	0.000670399	27211	Snord35a	ENSMUSG00000065818	small nucleolar RNA, C/D box 35A
4494.350	3.040	0.655	4.642	0.000003454	0.000884466	102465959	Mir6236	ENSMUSG00000098973	microRNA 6236
42.039	2.520	0.555	4.539	0.000005659	0.001150692	100217459	Snord70	NA	small nucleolar RNA, C/D box 70
138.574	2.685	0.595	4.515	0.000006330	0.001190163	27210	Snord34	ENSMUSG00000065878	small nucleolar RNA, C/D box 34
71.446	1.453	0.324	4.481	0.000007415	0.001336333	100217441	Snord23	ENSMUSG00000080478	small nucleolar RNA, C/D box 23
194.003	3.442	0.770	4.471	0.000007784	0.001376430	102466620	Mir6240	ENSMUSG00000098343	microRNA 6240
68.462	1.776	0.403	4.405	0.000010568	0.001706231	100302529	Snord72	NA	small nucleolar RNA, C/D box 72
162.184	1.219	0.283	4.305	0.000016724	0.002278893	100217414	Scarna3a	ENSMUSG00002074853	small Cajal body-specific RNA 3A
318.200	2.573	0.608	4.229	0.000023461	0.002800449	80828	Snord82	ENSMUSG00000064823	small nucleolar RNA, C/D box 82
186.481	2.801	0.672	4.170	0.000030438	0.003252850	219148	Fam167a	ENSMUSG00000035095	family with sequence similarity 167 member A
15.424	3.105	0.749	4.148	0.000033557	0.003461271	330737	5430403N17Rik	ENSMUSG00000109761	RIKEN cDNA 5430403N17 gene
229.439	2.302	0.557	4.135	0.000035508	0.003513555	100217443	Snord12	NA	small nucleolar RNA, C/D box 12
560.671	2.502	0.610	4.098	0.000041611	0.003936569	266793	Snord87	ENSMUSG00000093178	small nucleolar RNA, C/D box 87
8498.512	2.416	0.591	4.092	0.000042844	0.004027572	19799	Rn4.5s	NA	rRNA
126.730	1.132	0.278	4.070	0.000047058	0.004210517	100217442	Snora30	ENSMUSG00000065259	small nucleolar RNA, H/ACA box 30
166.851	2.983	0.739	4.038	0.000053851	0.004640532	102465895	Mir3535	ENSMUSG00000104627	microRNA 3535
271.768	1.774	0.442	4.012	0.000060198	0.004939898	27209	Snord32a	ENSMUSG00000065219	small nucleolar RNA, C/D box 32A
18.254	5.724	1.431	4.001	0.000063053	0.005145723	16492	Kcna4	ENSMUSG00000042604	potassium voltage-gated channel subfamily A member 4
185.837	2.164	0.559	3.872	0.000108003	0.007258674	100302565	Snord68	NA	small nucleolar RNA, C/D box 68
72.199	1.015	0.265	3.832	0.000127156	0.007869344	667214	9930111J21Rik1	ENSMUSG00000069893	

Up-regulated in Tert-CreER/flox

baseMean	log2 Fold Change	lfcSE	stat	pvalue	padj	entrez	gene_symbol	ensembl	
257.941	-1.885	0.167	-11.268	0.000000000	0.000000000	100040852	Gm3002	NA	alpha-takusan pseudogene
216.733	-1.427	0.194	-7.363	0.000000000	0.000000001	544990	Gm5795	ENSMUSG00000079409	predicted gene 5795
148.961	-1.377	0.202	-6.810	0.000000000	0.000000029	100041774	Gm10413	ENSMUSG00000095533	predicted gene 10413
158.082	-1.330	0.225	-5.908	0.000000003	0.000006424	56631	Trim17	ENSMUSG00000036964	tripartite motif containing 17
220.218	-1.268	0.215	-5.911	0.000000003	0.000006424	626415	4930467E23Rik	ENSMUSG00000096265	
190.802	-1.055	0.184	-5.727	0.000000010	0.000015201	56734	Tulp2	ENSMUSG00000023467	TUB like protein 2
101.658	-1.573	0.277	-5.679	0.000000014	0.000018325	68265	Iqcf3	ENSMUSG00000023577	IQ motif containing F3
55.342	-1.694	0.306	-5.534	0.000000031	0.000035844	545007	Gm5796	ENSMUSG00000096775	predicted gene 5796
77.734	-1.601	0.294	-5.443	0.000000052	0.000045737	73376	Tex33	ENSMUSG00000062154	testis expressed 33
190.450	-1.175	0.216	-5.452	0.000000050	0.000045737	22116	Tsks	ENSMUSG00000059891	testis specific serine kinase substrate
124.340	-1.392	0.257	-5.415	0.000000061	0.000050574	76718	Catsperg2	ENSMUSG00000049123	cation channel sperm associated auxiliary subunit gamma 2
227.287	-1.326	0.247	-5.377	0.000000076	0.000059108	100040599	Gm15319	ENSMUSG00000074449	
138.572	-1.162	0.219	-5.305	0.000000113	0.000076199	330010	Ttll10	ENSMUSG00000029074	tubulin tyrosine ligase like 10
210.380	-1.248	0.238	-5.237	0.000000163	0.000100898	100041530	Gm10409	ENSMUSG00000096488	predicted gene 10409
111.452	-1.393	0.267	-5.211	0.000000188	0.000110619	66755	4933415F23Rik	ENSMUSG00000073730	
294.526	-1.137	0.220	-5.176	0.000000227	0.000124703	18285	Odf1	ENSMUSG00000061923	outer dense fiber of sperm tails 1
84.815	-1.471	0.289	-5.085	0.000000367	0.000187806	74053	Grip1	ENSMUSG00000034813	glutamate receptor interacting protein 1
289.500	-1.014	0.202	-5.022	0.000000510	0.000244422	632687	March10	ENSMUSG00000078627	membrane associated ring-CH-type finger 10
152.558	-1.351	0.269	-5.015	0.000000530	0.000245898	58251	Cep295nl	ENSMUSG00000076433	CEP295 N-terminal like
108.929	-1.387	0.278	-4.987	0.000000614	0.000276499	114875	Plcz1	ENSMUSG00000030230	phospholipase C zeta 1
139.016	-1.037	0.209	-4.950	0.000000742	0.000315009	66720	Klhl10	ENSMUSG00000001558	kelch like family member 10
301.062	-1.103	0.223	-4.936	0.000000797	0.000328990	320827	C530008M17Rik	ENSMUSG00000036377	
121.978	-2.418	0.504	-4.798	0.000001605	0.000529596	100041874	Gm3558	ENSMUSG00000079364	
161.147	-1.197	0.250	-4.784	0.000001717	0.000547141	14625	Gykl1	ENSMUSG00000053624	glycerol kinase-like 1
293.548	-1.083	0.227	-4.778	0.000001768	0.000547141	210145	Irgc1	ENSMUSG00000062028	immunity-related GTPase family, cinema 1
144.584	-1.160	0.243	-4.773	0.000001817	0.000550782	22240	Dpysl3	ENSMUSG00000024501	dihydropyrimidinase like 3
204.176	-1.263	0.265	-4.767	0.000001868	0.000554927	70069	H1fnt	ENSMUSG00000048077	H1.7 linker histone
178.595	-1.153	0.244	-4.734	0.000002204	0.000629558	12661	Chl1	ENSMUSG00000030077	cell adhesion molecule L1 like
70.391	-1.685	0.363	-4.642	0.000003445	0.000884466	70902	Lpcat2b	ENSMUSG00000033794	lysophosphatidylcholine acyltransferase 2B
77.565	-1.368	0.296	-4.626	0.000003723	0.000930487	381229	Cfap58	ENSMUSG00000046585	cilia and flagella associated protein 58
162.146	-1.181	0.256	-4.617	0.000003885	0.000946051	73470	Kif2b	ENSMUSG00000046755	kinesin family member 2B
34.368	-1.822	0.396	-4.605	0.000004131	0.000989667	12824	Col2a1	ENSMUSG00000022483	collagen type II alpha 1 chain
69.336	-1.535	0.336	-4.572	0.000004819	0.001046485	79459	Aldoart2	ENSMUSG00000063129	aldolase 1 A, retrogene 2
202.092	-1.218	0.267	-4.571	0.000004845	0.001046485	217341	Qrich2	ENSMUSG00000070331	glutamine rich 2
112.544	-1.268	0.280	-4.531	0.000005882	0.001150692	20503	Slc16a7	ENSMUSG00000020102	solute carrier family 16 member 7
132.787	-1.113	0.246	-4.530	0.000005888	0.001150692	74052	Ttc21a	ENSMUSG00000032514	tetratricopeptide repeat domain 21A
110.936	-1.313	0.291	-4.516	0.000006288	0.001190163	69380	1700013G24Rik	ENSMUSG00000041399	
87.913	-1.091	0.242	-4.516	0.000006309	0.001190163	213272	Txndc2	ENSMUSG00000050612	thioredoxin domain containing 2
97.798	-1.413	0.315	-4.480	0.000007468	0.001336333	11636	Ak1	ENSMUSG00000026817	adenylate kinase 1
276.721	-1.043	0.233	-4.483	0.000007375	0.001336333	73472	Spata18	ENSMUSG00000029155	spermatogenesis associated 18
97.850	-1.465	0.329	-4.459	0.000008229	0.001437940	74288	Spem1	ENSMUSG00000041165	spermatid maturation 1
147.507	-1.338	0.301	-4.448	0.000008679	0.001465368	319634	Efcab5	ENSMUSG00000050944	EF-hand calcium binding domain 5
143.211	-1.165	0.262	-4.440	0.000009013	0.001504088	231045	4931409K22Rik	ENSMUSG00000038199	
253.690	-1.065	0.242	-4.401	0.000010798	0.001724513	69439	Mroh4	ENSMUSG00000022603	maestro heat-like repeat family member 4
76.623	-1.188	0.271	-4.390	0.000011320	0.001738202	12845	Comp	ENSMUSG00000031849	cartilage oligomeric matrix protein
74.295	-1.288	0.294	-4.382	0.000011769	0.001783789	231201	AF366264	ENSMUSG00000057116	SUMO/sentrin specific peptidase 2-like 2A
433.780	-1.271	0.294	-4.326	0.000015157	0.002179830	100861668	Gm21119	ENSMUSG00000095294	
134.238	-1.226	0.284	-4.324	0.000015348	0.002179830	69339	Ccdc54	ENSMUSG00000050685	coiled-coil domain containing 54
209.039	-1.007	0.233	-4.329	0.000014997	0.002179830	14571	Gpd2	ENSMUSG00000026827	glycerol-3-phosphate dehydrogenase 2
120.561	-1.221	0.283	-4.318	0.000015772	0.002210067	100041678	Gm3500	ENSMUSG00000096003	
55.233	-1.352	0.315	-4.294	0.000017557	0.002332019	245865	Spag4	ENSMUSG00000038180	sperm associated antigen 4
106.814	-1.105	0.257	-4.296	0.000017411	0.002332019	67356	Tmco5	ENSMUSG00000027355	transmembrane and coiled-coil domains 5
52.829	-1.655	0.388	-4.267	0.000019839	0.002562273	73968	4930444F02Rik	NA	
55.097	-1.492	0.350	-4.260	0.000020402	0.002612317	108161	Fam50b	ENSMUSG00000038246	family with sequence similarity 50 member B
83.613	-1.417	0.334	-4.243	0.000022089	0.002712380	239591	Ttll8	ENSMUSG00000022388	tubulin tyrosine ligase like 8
43.198	-1.351	0.318	-4.244	0.000021989	0.002712380	554327	2610042L04Rik	ENSMUSG00000079388	
59.387	-1.219	0.287	-4.243	0.000022075	0.002712380	243538	Cfap100	ENSMUSG00000048794	cilia and flagella associated protein 100
129.132	-1.079	0.254	-4.243	0.000022096	0.002712380	243822	Fam71e2	ENSMUSG00000092518	golgi associated RAB2 interactor family member 5B
54.886	-1.639	0.388	-4.228	0.000023529	0.002800449	71281	Apobec4	ENSMUSG00000055547	apolipoprotein B mRNA editing enzyme catalytic polypeptide like 4
69.464	-1.091	0.258	-4.228	0.000023568	0.002800449	432825	Gm5458	ENSMUSG00000095024	
48.275	-1.301	0.308	-4.224	0.000024031	0.002832831	66772	Asb17	ENSMUSG00000038997	ankyrin repeat and SOCS box-containing 17
149.109	-1.016	0.241	-4.216	0.000024847	0.002877301	70821	4921507P07Rik	ENSMUSG00000029828	
103.496	-1.123	0.267	-4.207	0.000025933	0.002962934	100041515	Gm3383	ENSMUSG00000096629	
34.897	-1.386	0.330	-4.195	0.000027310	0.003073046	67620	Lrp2bp	ENSMUSG00000031637	LRP2 binding protein
91.578	-1.353	0.324	-4.179	0.000029267	0.003244026	237958	Sppl2c	ENSMUSG00000049506	signal peptide peptidase like 2C
89.152	-1.436	0.345	-4.168	0.000030735	0.003260778	70897	Fam71d	ENSMUSG00000056987	golgi associated RAB2 interactor family member 2
16.957	-2.987	0.718	-4.159	0.000031908	0.003361168	NA	Smok4a	ENSMUSG00000079711	sperm motility kinase 4A
60.561	-1.521	0.368	-4.133	0.000035733	0.003513555	70952	Poteg	ENSMUSG00000063932	POTE ankyrin domain family member G
80.042	-1.422	0.344	-4.138	0.000035075	0.003513555	75015	Samd13	ENSMUSG00000048652	sterile alpha motif domain containing 13
90.771	-1.369	0.331	-4.137	0.000035256	0.003513555	22114	Tssk1	ENSMUSG00000041566	testis-specific serine kinase 1
80.303	-1.206	0.292	-4.135	0.000035502	0.003513555	224814	Abcc10	ENSMUSG00000032842	ATP binding cassette subfamily C member 10
173.103	-1.106	0.267	-4.136	0.000035391	0.003513555	56523	Pmfbp1	ENSMUSG00000031727	polyamine modulated factor 1 binding protein 1
62.750	-1.298	0.314	-4.129	0.000036372	0.003530898	73523	Pebp4	ENSMUSG00000022085	phosphatidylethanolamine binding protein 4
25.512	-1.677	0.408	-4.106	0.000040272	0.003859089	74472	Stpg3	ENSMUSG00000036770	sperm-tail PG-rich repeat containing 3
79.232	-1.309	0.319	-4.103	0.000040863	0.003890624	53857	Tuba8	ENSMUSG00000030137	tubulin alpha 8
79.238	-1.582	0.387	-4.084	0.000044264	0.004109079	75886	Gstt4	ENSMUSG00000009093	glutathione S-transferase theta 4
126.321	-1.260	0.309	-4.076	0.000045864	0.004147851	20849	Stat4	ENSMUSG00000062939	signal transducer and activator of transcription 4
88.382	-1.230	0.302	-4.075	0.000046078	0.004147851	227998	4933409G03Rik	ENSMUSG00000053896	
116.294	-1.089	0.267	-4.078	0.000045405	0.004147851	243897	Ggn	ENSMUSG00000031493	gametogenetin
106.091	-1.004	0.249	-4.038	0.000053826	0.004640532	75721	4932414N04Rik	ENSMUSG00000079324	
83.814	-1.301	0.323	-4.027	0.000056377	0.004738099	66337	Fam229b	ENSMUSG00000051736	family with sequence similarity 229 member B
120.210	-1.182	0.294	-4.022	0.000057707	0.004788379	67722	Actl11	ENSMUSG00000066368	actin-like 11
63.719	-1.512	0.378	-3.995	0.000064667	0.005248589	78469	Tmem247	ENSMUSG00000037689	transmembrane protein 247
160.386	-1.048	0.263	-3.993	0.000065208	0.005263740	100039043	Gm10731	NA	predicted gene 10731
370.212	-1.330	0.334	-3.981	0.000068498	0.005462705	11642	Akap3	ENSMUSG00000030344	A-kinase anchoring protein 3
116.184	-1.079	0.271	-3.980	0.000068776	0.005462705	386611	Rnf133	ENSMUSG00000051956	ring finger protein 133

94.925	-1.271	0.322	-3.951	0.000077706	0.006042783	18573	Pde1a	ENSMUSG00000059173	phosphodiesterase 1A
61.576	-1.564	0.396	-3.949	0.000078461	0.006069704	73412	Nme8	ENSMUSG00000041138	NME/NM23 family member 8
50.910	-1.282	0.325	-3.940	0.000081382	0.006245506	75732	Iqcd	ENSMUSG00000029601	IQ motif containing D
92.838	-1.192	0.303	-3.931	0.000084463	0.006304194	238662	Spata31d1b	ENSMUSG00000091311	spermatogenesis associated 31 subfamily D, member 1B
51.275	-1.510	0.384	-3.928	0.000085497	0.006317859	329366	Ccdc187	ENSMUSG00000048038	coiled-coil domain containing 187
52.541	-1.385	0.354	-3.917	0.000089635	0.006431621	72219	Spata31d1a	ENSMUSG00000050876	spermatogenesis associated 31 subfamily D, member 1A
38.944	-1.587	0.406	-3.903	0.000094879	0.006678856	73368	Col20a1	ENSMUSG00000016356	collagen type XX alpha 1 chain
52.877	-1.384	0.355	-3.900	0.000096360	0.006719440	382077	Ccdc33	ENSMUSG00000037716	coiled-coil domain containing 33
113.573	-1.102	0.283	-3.891	0.000099808	0.006873480	100041735	Gm3488	ENSMUSG00000095295	
113.573	-1.102	0.283	-3.891	0.000099808	0.006873480	666329	Gm3317	ENSMUSG00000095912	predicted gene 3317
84.650	-1.027	0.264	-3.891	0.000099958	0.006873480	73344	1700034J05Rik	ENSMUSG00000040163	
109.519	-1.215	0.315	-3.857	0.000114849	0.007384650	70881	Nt5c1b	ENSMUSG00000020622	5'-nucleotidase, cytosolic IB
118.787	-1.159	0.300	-3.858	0.000114454	0.007384650	280668	Adam1a	ENSMUSG00000072647	ADAM metallopeptidase domain 1A (pseudogene)
149.232	-1.059	0.274	-3.862	0.000112313	0.007384650	59010	Sqor	ENSMUSG00000005803	sulfide quinone oxidoreductase
105.275	-1.256	0.326	-3.853	0.000116563	0.007428117	229776	Cdc14a	ENSMUSG00000033502	cell division cycle 14A
96.434	-1.133	0.294	-3.852	0.000117025	0.007428117	76378	Ropn1	ENSMUSG00000022832	rhophilin associated tail protein 1
86.491	-1.175	0.306	-3.844	0.000120934	0.007579032	330277	Fam71f1	ENSMUSG00000039742	golgi associated RAB2 interactor 1B
78.033	-1.313	0.343	-3.827	0.000129500	0.007981166	100286843	Gm16325	ENSMUSG00000085810	
76.911	-1.366	0.357	-3.823	0.000132064	0.008105559	18802	Plcd4	ENSMUSG00000026173	phospholipase C delta 4
54.719	-1.363	0.357	-3.817	0.000134865	0.008243413	71868	Thegl	ENSMUSG00000029248	theg spermatid protein like
141.050	-1.014	0.267	-3.794	0.000148104	0.008862018	320225	Catsperg1	ENSMUSG00000049676	cation channel sperm associated auxiliary subunit gamma 1
211.838	-1.473	0.390	-3.773	0.000161441	0.009366749	27222	Atp1a4	ENSMUSG00000007107	ATPase Na+/K+ transporting subunit alpha 4
34.853	-1.431	0.379	-3.776	0.000159413	0.009366749	320118	Fbxl13	ENSMUSG00000048520	F-box and leucine rich repeat protein 13
88.811	-1.389	0.368	-3.774	0.000160591	0.009366749	245884	Fam71f2	ENSMUSG00000079652	golgi associated RAB2 interactor 1A
64.749	-1.353	0.358	-3.775	0.000159721	0.009366749	68274	Toporsl	ENSMUSG00000028314	topoisomerase I binding, arginine/serine-rich like
46.886	-1.241	0.329	-3.777	0.000158668	0.009366749	231287	Atp10d	ENSMUSG00000046808	ATPase phospholipid transporting 10D (putative)
140.472	-1.047	0.278	-3.766	0.000166104	0.009525658	385668	Lca5l	ENSMUSG00000045275	lebercilin LCA5 like
55.333	-1.335	0.355	-3.762	0.000168426	0.009584817	13137	Cd55b	ENSMUSG00000026401	CD55 molecule, decay accelerating factor for complement B
82.343	-1.211	0.322	-3.763	0.000168028	0.009584817	213980	Fbxw10	ENSMUSG00000090173	F-box and WD repeat domain containing 10
48.261	-1.825	0.486	-3.752	0.000175251	0.009897355	67320	Iqcf4	ENSMUSG00000041009	IQ motif containing F4

Hasegawa et al

SI table 2: RT-PCR primers used in this study

Gene	Primer1	Primer2	Detection
Actb	GGCTGTATTCCCCTCCATCG	CCAGTTGGTAACAATGCCATGT	SYBR
Gfra1	GCACCAAGTACCGCACACT	GCGGCAGTTGTAGAGAGACTTC	SYBR
Ngn3	AATGATCGGGAGCGCAATCG	CGCAGGGTCTCGATCTTTG	SYBR
Kit	GCCACGTCTCAGCCATCTG	GTCGCCAGCTTCAACTATTAACT	SYBR
Tert exon2	TGAAAGTAGAGGATTGCCACTG	CCTCAGACGGTGCTCTGC	Universal ProbeLibrary Probe #66
Tert exon6	TTCACCCAGCGTCTCAAGAC	CCCGGTCACATCTGCCTTAA	Universal ProbeLibrary Probe #93
mouse Myc	CCTAGTGCTGCATGAGGAGA	TCCACAGACACCACATCAATTT	Universal ProbeLibrary Probe #77
human MYC	GGCTCCTGGCAAAAGGTCA	CTGCGTAGTTGTGCTGATGT	SYBR

Hasegawa et al., SI Figure1

Extended data fig.3b
Southern blot, 5’ probe

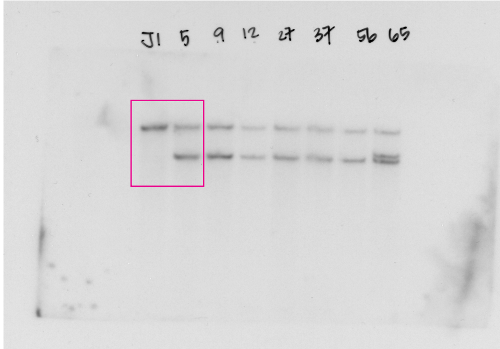

Extended data fig.3b
Southern blot, 3’ probe

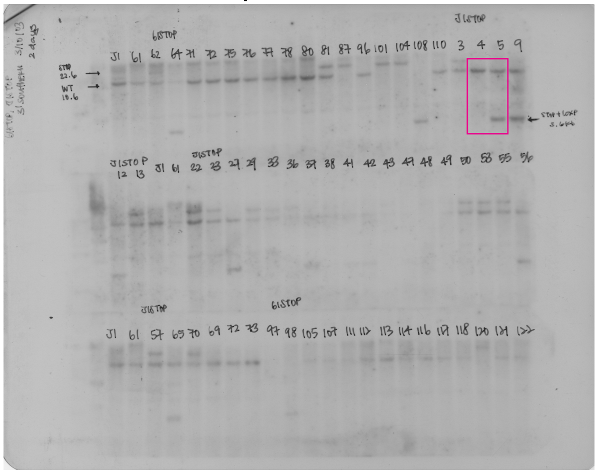

Extended data fig.3c, TRAP assay

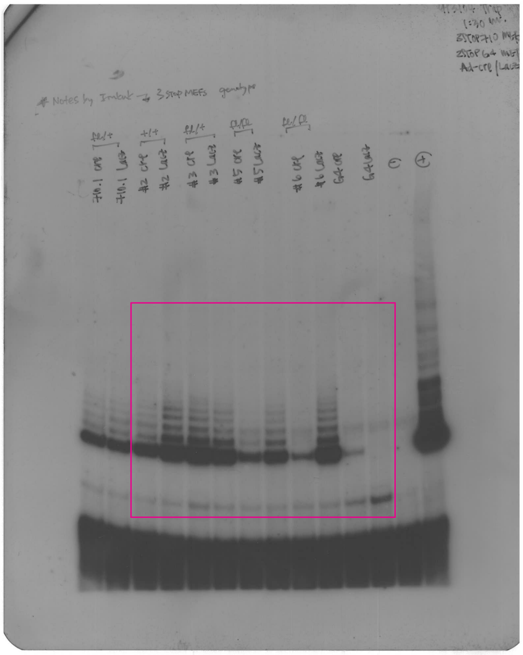

Extended data fig.3h, TRAP assay

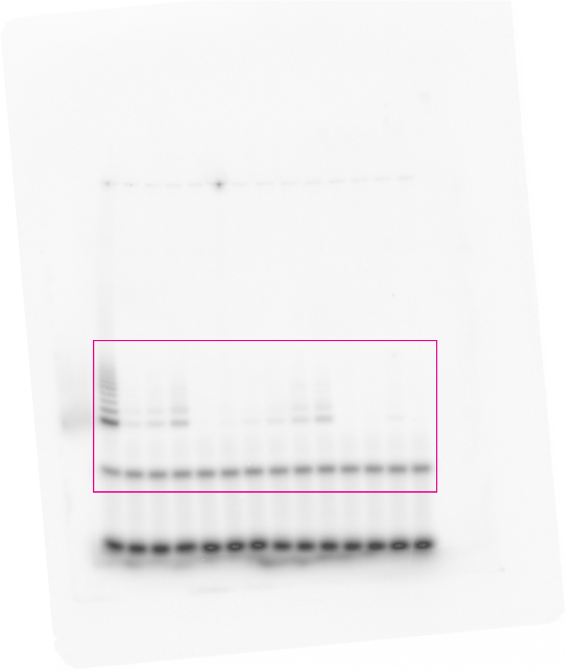

Extended data fig.3d, genotyping PCR

Hprt

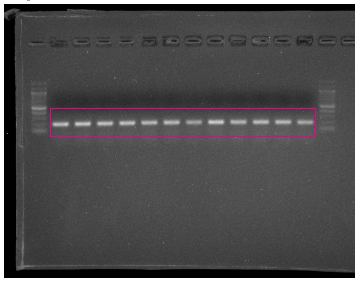

Tert-del

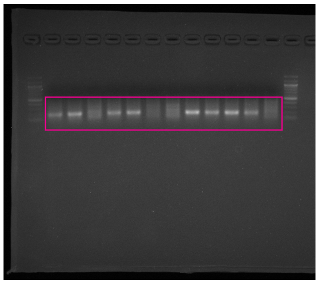

Hasegawa et al., SI Figure2

Extended data fig.4a, TRAP assay

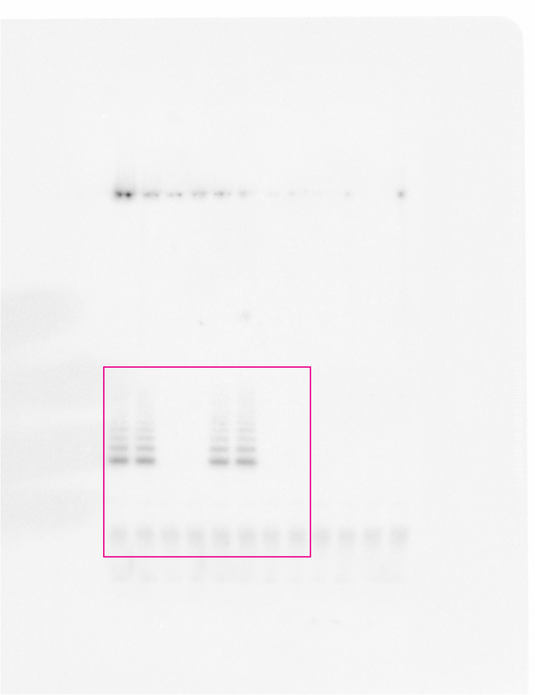

Extended data fig.4c, TRAP assay

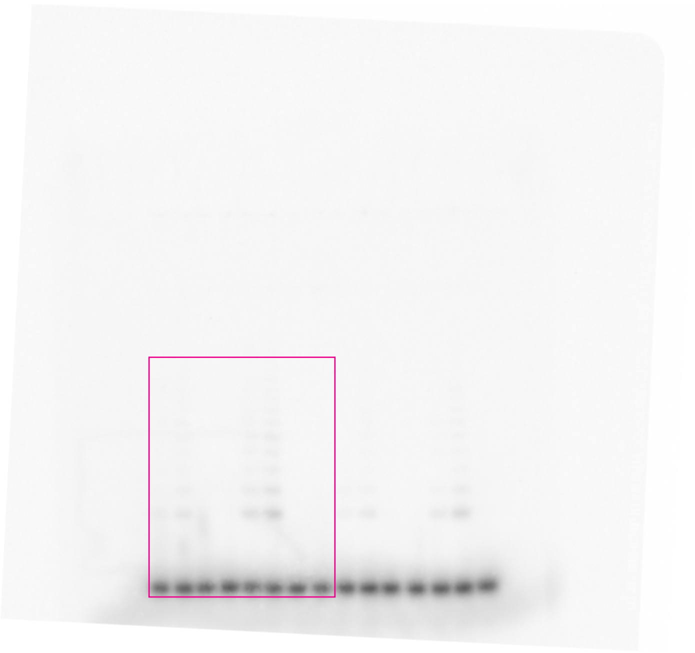

Hasegawa et al., SI figure3

a

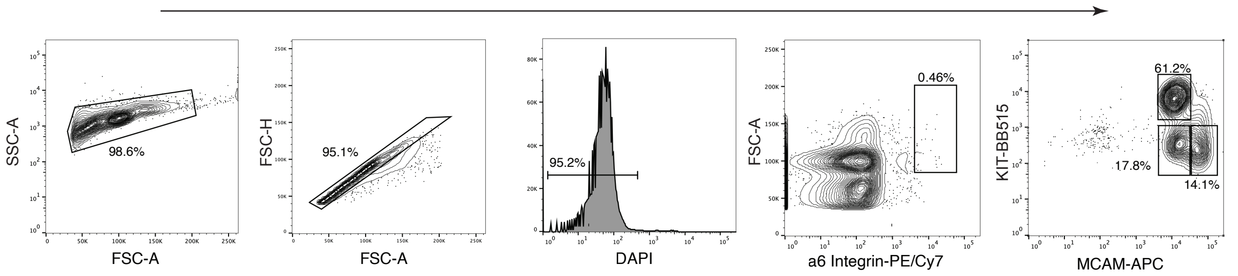

b

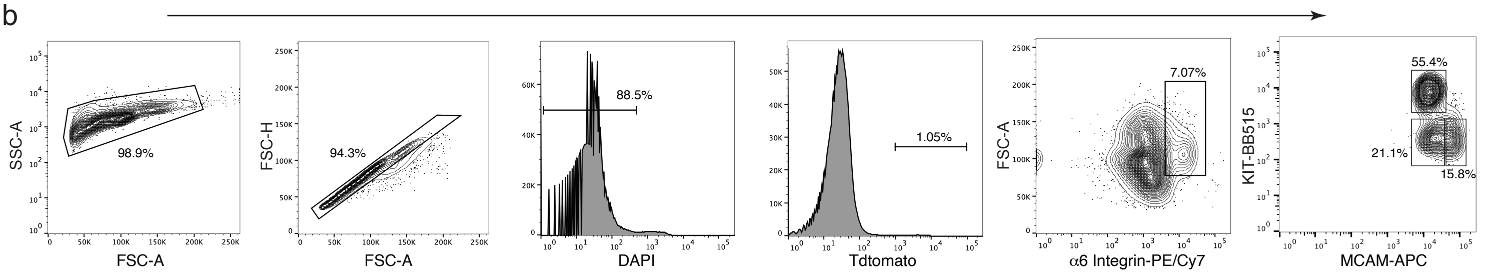

Gating strategy.

a) Gating strategy for Extended Data figure 1c,e. Cells were selected by scatter properties. Single cells were gated by the area and height of forward scatter. Viable cells were selected by DAPI exclusion. Spermatogonia were enriched by α6 Integrin. MCAM-h, MCAM-m, DS were selected by MCAM and KIT.

b) Gating strategy for Extended Data figure 6a. Cells were selected by scatter properties. Single cells were gated by the area and height of forward scatter. Viable cells were selected by DAPI exclusion. Labeled cells were gated by Tdtomato. Spermatogonia were enriched by α6 Integrin. MCAM-h, MCAM-m, DS were selected by MCAM and KIT.
