# Supplementary material for: Clonal inactivation of TERT impairs stem cell competition
Source: Nature. 2024 Jul 17;632(8023):201–8. doi: 10.1038/s41586-024-07700-w (PMC11291281; doi:10.1038/s41586-024-07700-w)
Supplement: Supplementary file 2 — Reporting Summary [file 41586_2024_7700_MOESM2_ESM.pdf]

nature research

Corresponding author(s): Steven E. Artandi

Last updated by author(s): May 24, 2024

Reporting Summary

Nature Research wishes to improve the reproducibility of the work that we publish. This form provides structure for consistency and transparency in reporting. For further information on Nature Research policies, see Authors & Referees and the Editorial Policy Checklist.

Statistics

For all statistical analyses, confirm that the following items are present in the figure legend, table legend, main text, or Methods section.

n/a	Confirmed	
☐	☒	The exact sample size (*n*) for each experimental group/condition, given as a discrete number and unit of measurement
☐	☒	A statement on whether measurements were taken from distinct samples or whether the same sample was measured repeatedly
☐	☒	The statistical test(s) used AND whether they are one- or two-sided<br>*Only common tests should be described solely by name; describe more complex techniques in the Methods section.*
☒	☐	A description of all covariates tested
☐	☒	A description of any assumptions or corrections, such as tests of normality and adjustment for multiple comparisons
☐	☒	A full description of the statistical parameters including central tendency (e.g. means) or other basic estimates (e.g. regression coefficient) AND variation (e.g. standard deviation) or associated estimates of uncertainty (e.g. confidence intervals)
☐	☒	For null hypothesis testing, the test statistic (e.g. *F*, *t*, *r*) with confidence intervals, effect sizes, degrees of freedom and *P* value noted<br>*Give P values as exact values whenever suitable.*
☒	☐	For Bayesian analysis, information on the choice of priors and Markov chain Monte Carlo settings
☒	☐	For hierarchical and complex designs, identification of the appropriate level for tests and full reporting of outcomes
☒	☐	Estimates of effect sizes (e.g. Cohen's *d*, Pearson's *r*), indicating how they were calculated

Our web collection on statistics for biologists contains articles on many of the points above.

Software and code

Policy information about availability of computer code

Data collection	Flow cytometry data was acquired using BD FACSDiva software v8.0; Immunofluorescence data was captured using Leica Application Suite AF; Immunohistochemistry data was captured using Leica LAS 4.2.
Data analysis	Fluorescent/IHC images were analyzed by Leica LAS AF, ImageJ,Telometer 3.0.5, and Adobe Photoshop CC or later versions. Statistics and plots were generated by ggplot2 in R and GraphPad Prism 8. Flowjo v9 was used for FACS data.

For manuscripts utilizing custom algorithms or software that are central to the research but not yet described in published literature, software must be made available to editors/reviewers. We strongly encourage code deposition in a community repository (e.g. GitHub). See the Nature Research guidelines for submitting code & software for further information.

Data

Policy information about availability of data

All manuscripts must include a data availability statement. This statement should provide the following information, where applicable:

- Accession codes, unique identifiers, or web links for publicly available datasets
- A list of figures that have associated raw data
- A description of any restrictions on data availability

All data that support the finding of this study is available in a publicly accessible repository. The source data for the RNA-seq and ATAC-seq study are available in the NCBI Gene Expression Omnibus (GEO) repository under accession number GSE14659. The resulting fastq files were aligned to the mouse reference genome (mm10). Source data are provided with this paper.

Field-specific reporting

Please select the one below that is the best fit for your research. If you are not sure, read the appropriate sections before making your selection.

☒ Life sciences ☐ Behavioural & social sciences ☐ Ecological, evolutionary & environmental sciences

For a reference copy of the document with all sections, see nature.com/documents/nr-reporting-summary-flat.pdf

Life sciences study design

All studies must disclose on these points even when the disclosure is negative.

Sample size	Sample sizes were set using power calculation, based on effect size, significant level and power level.
Data exclusions	No individual samples or animals were excluded from any analyses.
Replication	All the experiments were replicated more than two times and statistically analyzed.
Randomization	The animal were randomly assigned to each group (control/experimental).
Blinding	The investigators were not blinded to the identities of the samples. All experimental and corresponding control samples were collected and analyzed at the same time under the same condition. Quantification was made with the same software settings.

Reporting for specific materials, systems and methods

We require information from authors about some types of materials, experimental systems and methods used in many studies. Here, indicate whether each material, system or method listed is relevant to your study. If you are not sure if a list item applies to your research, read the appropriate section before selecting a response.

Materials & experimental systems

n/a	Involved in the study	
☐	☒	Antibodies
☒	☐	Eukaryotic cell lines
☒	☐	Palaeontology
☐	☒	Animals and other organisms
☒	☐	Human research participants
☒	☐	Clinical data

Methods

n/a	Involved in the study	
☒	☐	ChIP-seq
☐	☒	Flow cytometry
☒	☐	MRI-based neuroimaging

Antibodies

Antibodies used	The following antibodies were used in this study: anti-RFP (Abcam, ab124754, rabbit polyclonal, 1:500 dilution for section and whole IF without TSA or 1:3000 dilution for IF with TSA); anti-RFP (MBL, M208-3, mouse monoclonal 1G9 and 3G5, 1:200 dilution for whole IF); anti-Ecadherin (R&D systems, AF748, goat polyclonal, 1:200 dilution for whole IF); anti-BrdU (Bio-rad, MCA2483, mouse monoclonal Bu201, 1:500 dilution for IF); anti-GFRa1 (R&D systems, AF560, goat polyclonal, 1:200 dilution for whole and section IF); anti-KIT (Cell Signaling Technology, 3074, rabbit monoclonal D13A2, 1:200 dilution for whole IF); anti-PLZF (Santa Cruz, sc-22839, rabbit monoclonal H-300, 1:200 dilution for IF without TSA or 1:5000 dilution for IF with TSA); anti-cleaved PARP (Cell signaling, 9548, mouse monoclonal 7C9, 1:500 dilution for IF); anti-gH2AX (EMD Millipore, 05-636, mouse monoclonal JBW301, 1:2000 dilution for IF); anti-MYC (Cell Signaling Technology, 13987, rabbit monoclonal D3N8F, 1:500 dilution for IF); anti-a6 Integrin with Pe/Cy7 (Biolegend, 313622, rat monoclonal GoH3, 1:150 dilution for FC); anti-MCAM with APC (Biolegend, 134712, rat monoclonal ME-9F1, 1:200 dilution for FC); anti-KIT with BB515 (BD Biosciences, 564481, mouse monoclonal 2B8, 1:200 dilution for FC)
Validation	The validation information is available from Vendors. Anti-RFP (Abcam, ab124754), WB and IHC-p; anti-RFP (MBL, M208-3), FCM, ICC, IP, WB; anti-Ecadherin (R&D systems, AF748) WB, IHC, FC; anti-BrdU (Bio-rad, MCA2483; FC, IHC, IF; anti-GFRa1 (R&D systems, AF560) WB, IHC; anti-KIT (Cell Signaling Technology, 3074), WB IP, IF; anti-PLZF (Santa Cruz, sc-22839) WB, IP, IF, ELISA; anti-cleaved PARP (Cell signaling, 9548), WB; anti-gH2AX (EMD Millipore, 05-636), ICC, IF, WB, ChIP, IHC; anti-MYC (Cell Signaling Technology, 13987), WB, IF, ChIP, FC; anti-a6 Integrin with Pe/Cy7 (Biolegend, 313622), FC; anti-MCAM with APC (Biolegend, 134712), FC; anti-KIT with BB515 (BD Biosciences, 564481), FC. We also validated antibody specificity using negative and positive control samples.

Animals and other organisms

Policy information about studies involving animals; ARRIVE guidelines recommended for reporting animal research

Laboratory animals	Tert flox/+ mice were generated in J1 mouse ES cells. Rosa-lslTdtomato, Rosa-lsl-tTA, TetO-hMYC, Terc-KO, Trp53-flox mice were obtained from the Jackson Laboratory. Tert-CreER, TetO-Tert, TetO-Tertci, Tert-Tdtomato mice were previously reported. Two to four-month old mice of mixed background were used. Only males were used in this study.
Wild animals	No wild animals were used in this study.
Field-collected samples	No field collected samples were used in the study
Ethics oversight	All animal experiments were approved by the Administrative Panel on Laboratory Animal Care (APLAC) in protocol APLAC-12684 and all experiments were in compliance with the ethical regulations of Stanford University.

Note that full information on the approval of the study protocol must also be provided in the manuscript.

Flow Cytometry

Plots

Confirm that:

☒ The axis labels state the marker and fluorochrome used (e.g. CD4-FITC).

☒ The axis scales are clearly visible. Include numbers along axes only for bottom left plot of group (a 'group' is an analysis of identical markers).

☒ All plots are contour plots with outliers or pseudocolor plots.

☒ A numerical value for number of cells or percentage (with statistics) is provided.

Methodology

Sample preparation	Described in the Method sections.
Instrument	FACSAria 2
Software	BD FACSDiva software v8.0 and FlowJo.
Cell population abundance	1) Extended Data figure 1c,e, US-h: 058% of total events. US-m: 0.073% of total events. DS: 0.25% of total events. 2) Extended Data figure 6a, US-h: 0.0097% of total events, US-m of total events: 0.013%, DS: 0.034% of total events.
Gating strategy	1) Extended Data figure 1c,e. Cells were selected by scatter properties. Single cells were gated by the area and height of forward scatter. Viable cells were selected by DAPI exclusion. Spermatogonia were enriched by α6 Integrin. MCAM-h, MCAM-m, DS were selected by MCAM and KIT. 2) Extended Data figure 6a. Cells were selected by scatter properties. Single cells were gated by the area and height of forward scatter. Viable cells were selected by DAPI exclusion. Labeled cells were gated by Tdtomato. Spermatogonia were enriched by α6 Integrin. MCAM-h, MCAM-m, DS were selected by MCAM and KIT.

☒ Tick this box to confirm that a figure exemplifying the gating strategy is provided in the Supplementary Information.
